# Personalized Management and Treatment of Alzheimer’s Disease

**DOI:** 10.3390/life12030460

**Published:** 2022-03-21

**Authors:** Ramón Cacabelos, Vinogran Naidoo, Olaia Martínez-Iglesias, Lola Corzo, Natalia Cacabelos, Rocío Pego, Juan C. Carril

**Affiliations:** 1 Department of Genomic Medicine, International Center of Neuroscience and Genomic Medicine, EuroEspes Biomedical Research Center, 15165 Bergondo, Corunna, Spain; 2 Department of Neuroscience, International Center of Neuroscience and Genomic Medicine, EuroEspes Biomedical Research Center, 15165 Bergondo, Corunna, Spain; neurociencias@euroespes.com; 3 Department of Medical Epigenetics, International Center of Neuroscience and Genomic Medicine, EuroEspes Biomedical Research Center, 15165 Bergondo, Corunna, Spain; epigenetica@euroespes.com; 4 Department of Medical Biochemistry, International Center of Neuroscience and Genomic Medicine, EuroEspes Biomedical Research Center, 15165 Bergondo, Corunna, Spain; analisis@euroespes.com; 5 Department of Medical Documentation, International Center of Neuroscience and Genomic Medicine, EuroEspes Biomedical Research Center, 15165 Bergondo, Corunna, Spain; serviciodocumentacion@euroespes.com; 6 Department of Neuropsychology, International Center of Neuroscience and Genomic Medicine, EuroEspes Biomedical Research Center, 15165 Bergondo, Corunna, Spain; neuropsicologia@euroespes.com; 7 Department of Genomics and Pharmacogenomics, International Center of Neuroscience and Genomic Medicine, EuroEspes Biomedical Research Center, 15165 Bergondo, Corunna, Spain; genomica@euroespes.com

**Keywords:** Alzheimer’s disease, anti-dementia drugs, biomarkers, cerebrovascular genomics, concomitant disorders, neurodegenerative genomics, pathogenic genes, phenotype-modifying treatments, pharmacogenomics

## Abstract

Alzheimer’s disease (AD) is a priority health problem with a high cost to society and a large consumption of medical and social resources. The management of AD patients is complex and multidisciplinary. Over 90% of patients suffer from concomitant diseases and require personalized therapeutic regimens to reduce adverse drug reactions (ADRs), drug–drug interactions (DDIs), and unnecessary costs. Men and women show substantial differences in their AD-related phenotypes. Genomic, epigenetic, neuroimaging, and biochemical biomarkers are useful for predictive and differential diagnosis. The most frequent concomitant diseases include hypertension (>25%), obesity (>70%), diabetes mellitus type 2 (>25%), hypercholesterolemia (40%), hypertriglyceridemia (20%), metabolic syndrome (20%), hepatobiliary disorder (15%), endocrine/metabolic disorders (>20%), cardiovascular disorder (40%), cerebrovascular disorder (60–90%), neuropsychiatric disorders (60–90%), and cancer (10%). Over 90% of AD patients require multifactorial treatments with risk of ADRs and DDIs. The implementation of pharmacogenetics in clinical practice can help optimize the limited therapeutic resources available to treat AD and personalize the use of anti-dementia drugs, in combination with other medications, for the treatment of concomitant disorders.

## 1. Introduction

Alzheimer’s disease (AD) is a health priority in developed societies and in developing countries, along with cardiovascular disease, cancer, stroke, and major neuropsychiatric pathologies. Direct and indirect costs for the management of AD represent an overload on the economy of families, nations, and health resources. The worldwide cost of dementia exceeds US$800 billion dollars (>1% of GDP). The average cost per patient/year ranges from $30,000 to $60,000, depending on stage of the disease, quality of medical care, social status, and country (average cost in Europe: €32,506.73; in the United States: $49,781.74; in Japan: JPY5.95 million (approx. €30,000)) [1,2]. In terms of global costs (direct, indirect, and social costs, and costs of informal care), the World Health Organization (WHO), in 2019, estimated a total global societal cost of dementia of about US$1.3 trillion (>US$2.8 trillion by 2030) [3].

AD is the most prevalent form of dementia (50–60%). Vascular dementia (30–40%); other forms of dementia (10–15%); and mixed dementia, which is the most frequent form of dementia (>70%) in patients older than 75 years of age, are common presentations of dementia that follow AD in frequency. AD is more frequent in women than in men. The prevalence of dementia in males is 30.5/1000 and in females 48.2/1000 (probable AD, 11.7/1000 for males and 30.1/1000 for females) [4].

The phenotype of AD is the consequence of the premature death of neurons associated with genomic, epigenomic, cerebrovascular, and environmental factors. The clinical manifestation of dementia is characterized by progressive cognitive deterioration, behavioral changes, and functional decline [5,6,7,8]. 

Conventionally, two forms of AD are differentiated: an early form (early-onset AD, EOAD, <65 years) and a late-onset AD (LOAD, >65 years), within an apparent pathological continuum. EOAD is associated with familial forms of Mendelian genetics (familial AD, FAD), while LOAD shows a more complex pathogenesis in which a multitude of polymorphic variants in over 600 genes distributed throughout the human genome converge with diverse environmental factors, which attribute to the disease the false phenotypic profile of sporadic AD (sAD) [6,9]. 

Both forms of dementia exhibit common neuropathological hallmarks of amyloidopathy and tauopathy characterized by intracellular neurofibrillary tangles (NFTs), formed by hyperphosphorylation of tau protein in microtubules and neurofilaments, and extracellular deposits of aggregated β-amyloid (Aβ) in senile plaques and vessels (amyloid angiopathy), and probably exert synergistic effects on AD pathogenesis [10]. Dendritic dystrophy and desarborization, microglia activation, astrogliosis, and neuronal loss are also typical neuropathological markers in the hippocampus and neocortex, where neurotransmitter deficits (cholinergic, monoaminergic, glutamatergic, GABAergic, and neuropeptidergic), neurotrophic dysfunction, neuroinflammation, oxidative stress-related lipid peroxidation, and cerebrovascular (hypoperfusion) damage are also present [5,11,12,13].

The scientific community, the pharmaceutical industry, and the daily medical practice are facing important challenges for the management of dementia. The primary causes of AD and its pathogenic mechanisms are still unclear. Reliable biomarkers for an early diagnosis are not yet available. New drugs and novel therapeutic strategies able to slow-down or halt the course of the disease are urgent needs, assuming that present medications are inefficient and not cost-effective. Since the disease destroys the neurons of susceptible patients for decades before symptoms appear, the golden dream of AD scientific research would be to find a preventive remedy, administered in pre-symptomatic phases and capable of stopping the progressive destruction of the brain that leads to AD [14,15]. 

A further inescapable circumstance is to admit that the aging adult population accumulates many other pathologies concomitant with dementia that force the establishment of polypharmaceutical regimens, with the consequent increase in the risk of ADRs and dangerous drug–drug interactions (DDIs) [15,16]. In fact, over 80% of patients with dementia consume more than 10 different medications daily. Presently, the most efficient way to reduce ADRs and DDIs is to implement pharmacogenetic protocols for the personalization of pharmacological treatment of patients with dementia [17].

The objectives of this article are (i) to define the phenotypic profile of patients with dementia, using a large cohort of clinically characterized patients, from which we can infer concomitant pathologies and therapeutic needs, apart from conventional anti-dementia treatments, and (ii) to update the pharmacogenetics of anti-dementia drugs, as well as new forms of therapeutic intervention in AD.

## 2. Diagnostic Procedures

Although the diagnostic criteria for AD are relatively well-established by different scientific entities and expert groups [18,19,20], there is still a high rate of misdiagnoses and possible overdiagnosis of AD [21]. In highly specialized centers and in the selection and follow-up of patients undergoing clinical trials for the study of the safety and efficacy of anti-dementia drugs, we recommend a protocol comprising the following items: (i) clinical assessment (general, psychiatric, and neurologic), (ii) blood (biochemistry, hematology, metabolism, hormones, and neurotransmitters) and other body fluids (urine, cerebrospinal fluid) analyses, (iii) neuropsychological and psychometric assessment (cognition, mood, behavior, and motor function) with psychometric tools adapted and validated in each country), (iv) cardiovascular evaluation (ECG), (v) structural neuroimaging, (vi) functional neuroimaging, (vii) genetic screening (gene clusters of AD and cerebrovascular pathogenic genes), and (viii) pharmacogenetic profiling [13,22,23,24] (Table 1, Table 2, Table 3 and Table 4).

## 3. Phenotypic Features

In a cohort of randomly-selected patients diagnosed with AD (DSM-V and NINCDS-ADRDA criteria) (*n* = 2701; age: 67.63 ± 0.19 years; range: 50–96 years) of both sexes (1491 Females (55%); age: 68.26 ± 0.27 years; range: 50–96 years; 1210 Males (45%); age: 66.86 ± 0.28; *p* < 0.001), retrospectively recruited from the CIBE database (period: 2000–2020), we investigated sex-related common phenotypes, including biochemistry, hematology, metabolism, hormones, neurotransmitters, cardiovascular and cerebrovascular function, cognition, mood, behavior, and genomic and pharmacogenomic profiles (Table 1, Table 2, Table 3 and Table 4).

In terms of anthropometric parameters, substantial differences are observed between females and males in terms of height, weight, and body-mass index (BMI) (Table 1). Only 28% of patients show normal weight (29% F; 20% M), with obesity in >25% of the cases and only 1% with underweight (Table 1). 

Significant differences between females and males are present across many biological parameters, including (i) biochemical (glucose, total-cholesterol, HDL-cholesterol, LDL-cholesterol, triglycerides, urea, creatinine, uric acid, calcium, phosphorous, liver transaminases (ALAT, GGT), alkaline phosphatase, bilirubin, CPK, LDH, ions, iron, ferritin, folate, and vitamin B12), (ii) hormonal (TSH, PRL, ACTH, FSH, LH, estrogen, and testosterone), (iii) hematological (RCB, HCT, Hb, MCV, MCH, WBC, lymphocytes, monocytes, eosinophils, basophils, and platelets), (iv) cognitive, and (v) emotional markers. No differences are found in tumor markers (Table 1).

Cognitive markers (MMSE, ADAS) indicate than females show worse cognitive performance than males (Table 1). Cognitive impairment and depression are the first symptoms, which appear in over 90% of LOAD, and 80% and 9%, respectively, in EOAD cases [25].

Late-life depression is associated with cognitive impairment, and depression is linked with an increased risk for AD. Some overlapping pathogenic substrates (i.e., stress, cortisol levels, brain hypoperfusion, neuroinflammation, neurotrophic dysfunction, Aβ accumulation, tauopathic connections, epigenetic factors, and gut microbiota–brain axis) may explain the comorbidity of both clinical entities [26]. Mood disorders are more frequent in women than in men. Over 60% of AD patients show depressive symptoms, which are more severe in women than in men. Likewise, anxiety is also more frequent in females than in males. About 50% of men do not show anxiety, whereas only 30% of women with dementia are free of symptoms in early stages of the disease. Both anxiety and depression fluctuate with the clinical course of the disease [24,27,28,29,30]. Behavioral disorders and psychotic symptoms are also frequent (20–90%) in patients with AD along the clinical course of the disease [24,31,32].

**Figure 1:**
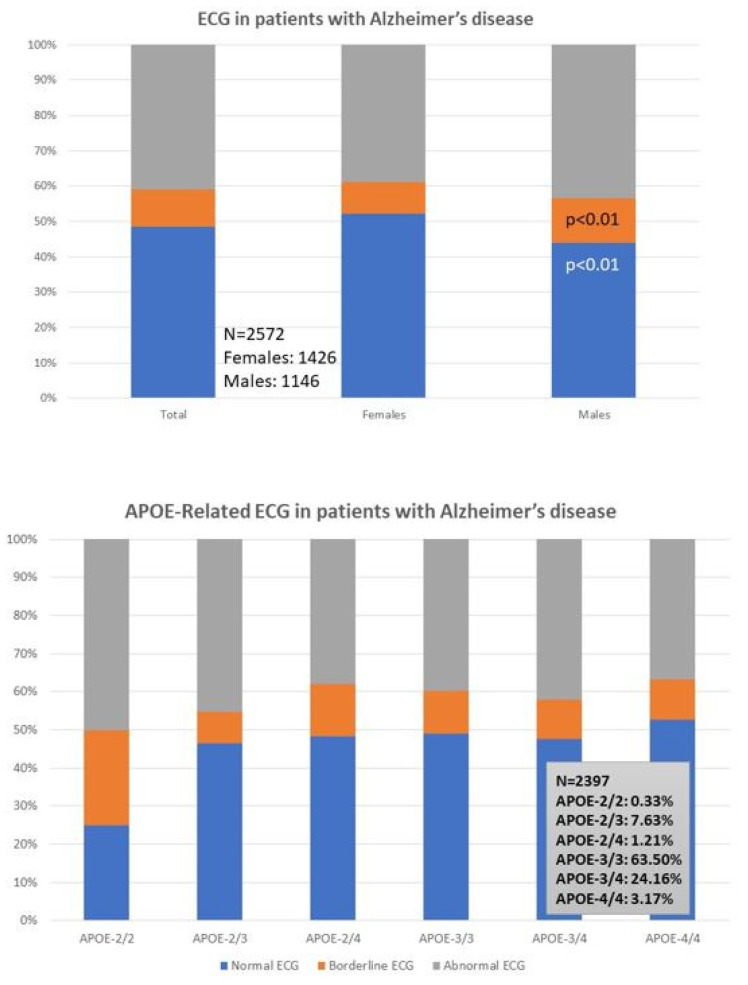
ECG (**upper panel**) and APOE-related ECG (**lower panel**) in patients with Alzheimer’s disease.

ECG is abnormal in 40% of the patients (38% F; 43% M). A normal ECG is found more frequently in females (52%) than males (43%; *p* < 0.01), and a borderline ECG appears more frequently in males (12%) than in females (9%; *p* < 0.01) (Table 1; Figure 1, upper panel); however, these differences are unrelated to apolipoprotein E (APOE) variants in both sexes (Figure 1, lower panel). Despite this global perception, and considering the small number of cases with the *APOE-2/2* and *APOE-4/4* genotypes, *APOE-4/4* carriers show abnormal ECGs (52%) more frequently than *APOE-3/3* carriers, especially in males (36%) as compared to females (10%); in contrast, homozygous *APOE-2/2* carriers exhibit the lowest frequency of abnormal ECG in the whole sample (25%), with twice the number of men (50%) showing abnormal ECG than *APOE-2/2* females (25%) (Figure 1, lower panel).

No sex-related differences are found in MRI anomalies (brain atrophy, leukoaraiosis, brain microinfarcts, and meningioma), which are present in over 70% of the cases (Table 1) [33,34,35,36].

## 4. Biomarkers

In addition to conventional clinical markers, which allow us to make a differential diagnosis and assess the possibility of concomitant diseases, the most useful biomarkers for a predictive diagnosis or diagnostic confirmation of antemortem AD are genomic markers, epigenetic biomarkers, neurotransmitters, and levels of Aβ/tau in the brain (PET Scan) and/or in body fluids [37,38,39,40,41,42,43,44,45,46]. 

### 4.1. Genomic Markers

Over 600 human genes are associated with AD [5,47,48]. Mutations in the amyloid precursor protein (*APP*) (>50 different mutations), presenilin 1 (*PSEN1* > 300 mutations), and presenilin-2 (*PSEN2* > 40 mutations) genes are present in a number of AD cases (5–10%), and induce brain amyloidopathy. Microtubule-associated protein tau (*MAPT*) gene mutations (>100), also present in some patients with AD, may cause brain tauopathies (e.g., frontotemporal dementia, Pick’s disease) [49,50,51]. Both conditions (amyloidopathy and tauopathy) are the two dominant pathogenic hypotheses in AD [52,53].

APP mutations can cause EOAD, with increased Aβ levels or Aβ fibrillogenesis, while some coding variants (*APP* A673T) may be protective with reduced Aβ levels [51]. EOAD dominant mutations tend to occur in the APP coding region or in the presenilin-related catalytic site of γ-secretase whose protease dysfunction is responsible for the abnormal process of APP cleavage and consequent accumulation of Aβ in senile plaques and vessels. In >40% AD cases, the presence of the apolipoprotein E4 (*APOE-4*) allele is the most important risk factor, involved in the impairment of Aβ clearance from brain tissue, in atherosclerosis and in hypoperfusion. Most immunotherapeutic interventions with Aβ antibodies (aducanumab, solanezumab, and crenezumab) attempt to halt the amyloidogenic process and slow-down cognitive deterioration in mild-AD or in presymptomatic cases with demonstrated genomic dysfunction [52]. In addition to these primary pathogenic genes, many other genes have been associated with AD in next-generation sequencing (NGS) and genome-wide association studies (GWAS) in different populations [47,48,54,55,56,57,58].

**Figure 2:**
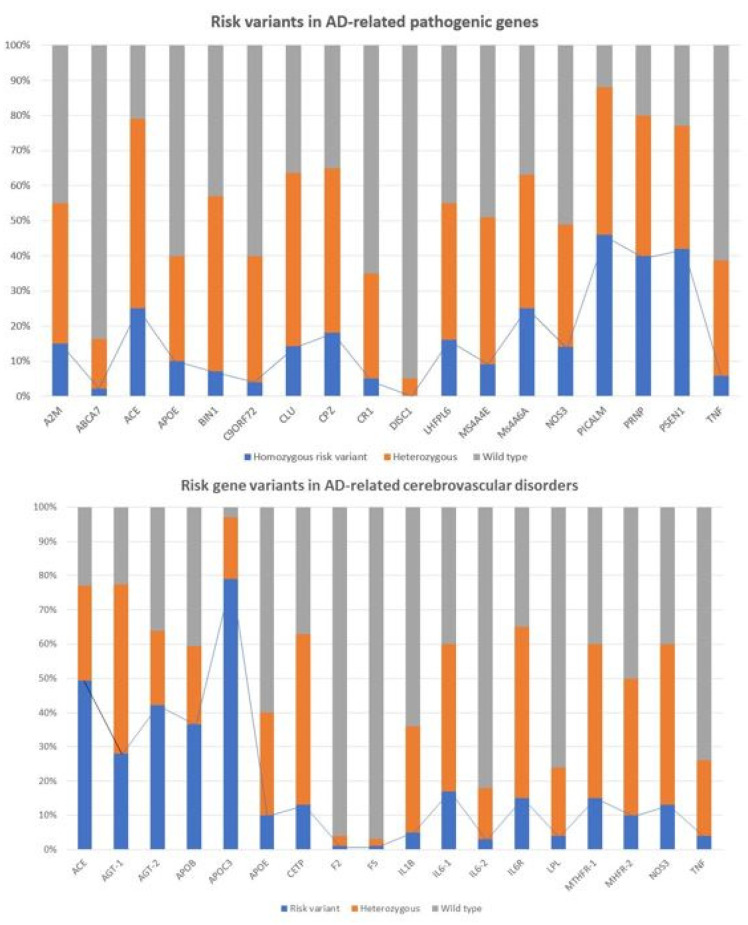
Pathogenic gene variants (**upper panel**) and cerebrovascular risk gene variants (**lower panel**) associated with Alzheimer’s disease. See Table 2 and Table 3 and Abbreviations for gene identification and SNPs of risk.

Recent studies show that multiple genetic defects can accumulate in the same case of AD, conditioning its phenotypic characteristics. In a genomic panel of 18 AD-related pathogenic genes (Table 2) and genotypes related to cerebrovascular disorders (Table 3; Figure 2), it can be verified that >60% of patients are carriers of more than 10 pathogenic variants.

**Figure 3:**
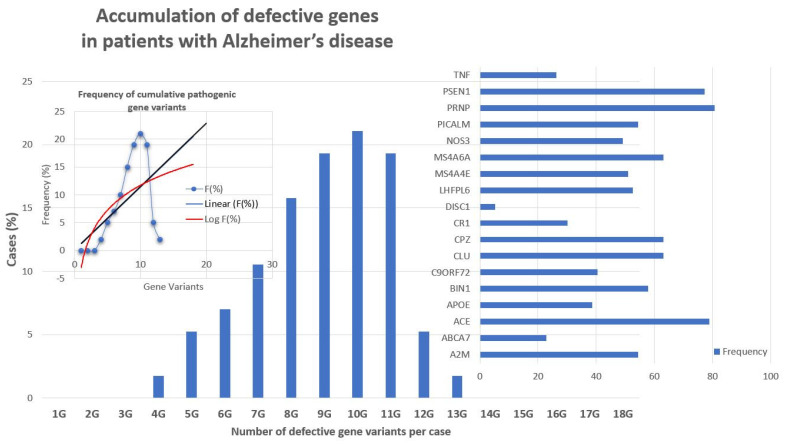
Accumulation of defective pathogenic gene variants in patients with Alzheimer’s disease.

The genes that most frequently accumulate pathogenic variants (>50%) in cases of AD are the following: *PRNP* (80.70%), *ACE* (78.94%), *PSEN1* (77.19%), *CLU* (63.15%), *CPZ* (63.15%), *MS4A6A* (63.15%), *BIN1* (57.89%), *A2M* (54.38%), *PICALM* (54.38%), *LHFPL6* (52.63%), and *MS4A4E* (50.87%) (Figure 3). 

The pathogenic load of *APOE-4* affects 35–40% of cases, with significant phenotypic consequences (Table 2, Table 3 and Table 4). *APOE-4/4* carriers tend to show an earlier age-at-onset in >80% of the cases; lower peripheral ApoE, nitric oxide, histamine, Aβ, HDL-cholesterol, and triglyceride levels; higher levels of total cholesterol and LDL-cholesterol; more pronounced brain atrophy and slower brain bioelectrical activity; more severe brain hemodynamic dysfunction represented by hypoperfusion, reduced brain blood flow velocity and increased pulsatility and resistance indices; increased lymphocyte apoptosis; faster cognitive deterioration; more frequent metabolic disorders, cardiovascular disorders, hypertension, atherosclerosis, liver metabolism dysfunction, behavioral disturbances, and alterations in circadian rhythm patterns; and a poor response to conventional treatments [5,11,12,13,22,48,59,60,61,62,63,64,65,66,67,68,69,70,71].

### 4.2. Epigenetic Markers

Various epigenetic aberrations are associated with AD pathogenesis, including hypomethylation/hypermethylation in the promoters of pathogenic genes, alterations in histones, and changes in the linear and three-dimensional structure of nuclear chromatin, as well as profound alterations in microRNAs (miRNAs) that regulate gene expression in the cellular cytoplasm. Some of these epigenetic alterations have been proposed as potential biomarkers of AD [72,73,74,75,76,77,78,79].

**Figure 4:**
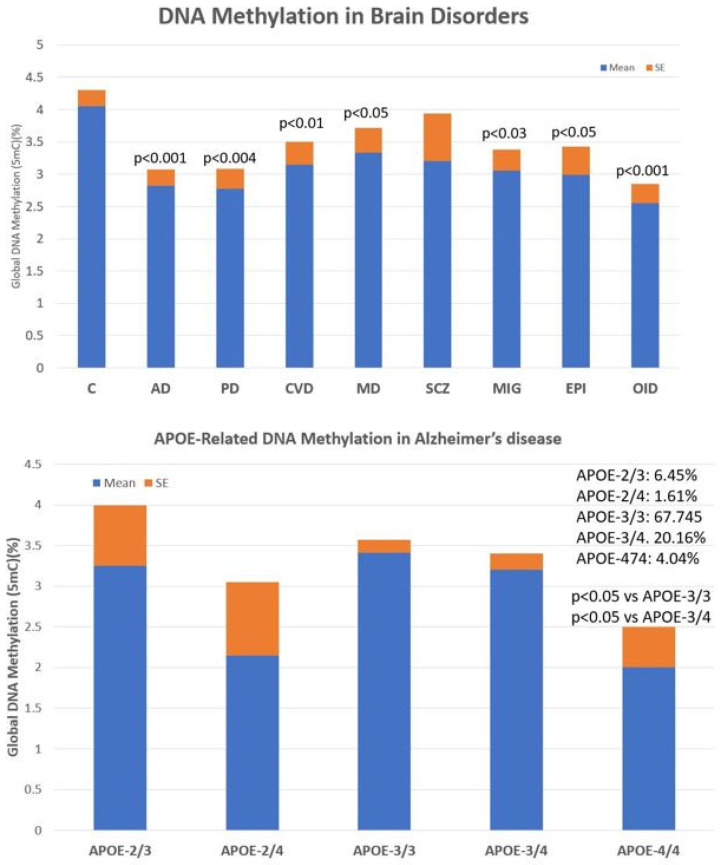
Global DNA methylation in patients with central nervous system disorders (**upper panel**) and APOE-related DNA methylation in patients with Alzheimer’s disease (**lower panel**). C: Control; AD: Alzheimer’s disease; PD: Parkinson’s disease; CVD: Cerebrovascular disorder; MD: Major Depression; SCZ: Schizophrenia and psychotic syndromes; MIG: Migraine; EPI: Epilepsy; OID: Organic Intellectual Disability.

The main problems observed with the use of epigenetic biomarkers in AD are their variability and lack of specificity. Changes in global DNA methylation are very sensitive and appear diminished in a multitude of central nervous system (CNS) diseases, such as AD (*p* < 0.001), Parkinson’s disease (*p* < 0.004), cerebrovascular disorders and stroke (*p* < 0.01), major depression (*p* < 0.05), migraine (*p* < 0.03), epilepsy (*p* < 0.05), and intellectual organic disability (OID) (*p* < 0.001), and to a lesser extent in schizophrenia (Figure 4, upper panel). These values are very sensitive to therapeutic interventions but unreliable as predictive or diagnostic values. The low diagnostic value of DNA methylation is compensated for by the exquisite sensitivity of this biomarker that responds in a highly sensitive manner to the therapeutic response of each patient. In addition, what appears to be important in AD is that global DNA methylation shows an *APOE*-dependent pattern. *APOE-4* carriers tend to show a more severe DNA hypomethylation pattern than patients carrying the *APOE-3* allele, which is aggravated in parallel with the degree of cognitive impairment (Figure 4, lower panel).

Epigenetic biomarkers can also help in the personalization of anti-AD treatments (Pharmacoepigenetics) [80,81,82,83] and serve as a guide in the search for epigenetic drugs with prophylactic and/or therapeutic action in the treatment of AD [72,81,82,83].

### 4.3. Neurotransmitters

Conventionally, the main neurotransmitter affected in AD is acetylcholine. However, premature neuronal death alters the levels of many other essential neurotransmitters for the normal functioning of the CNS [84,85,86].

**Figure 5:**
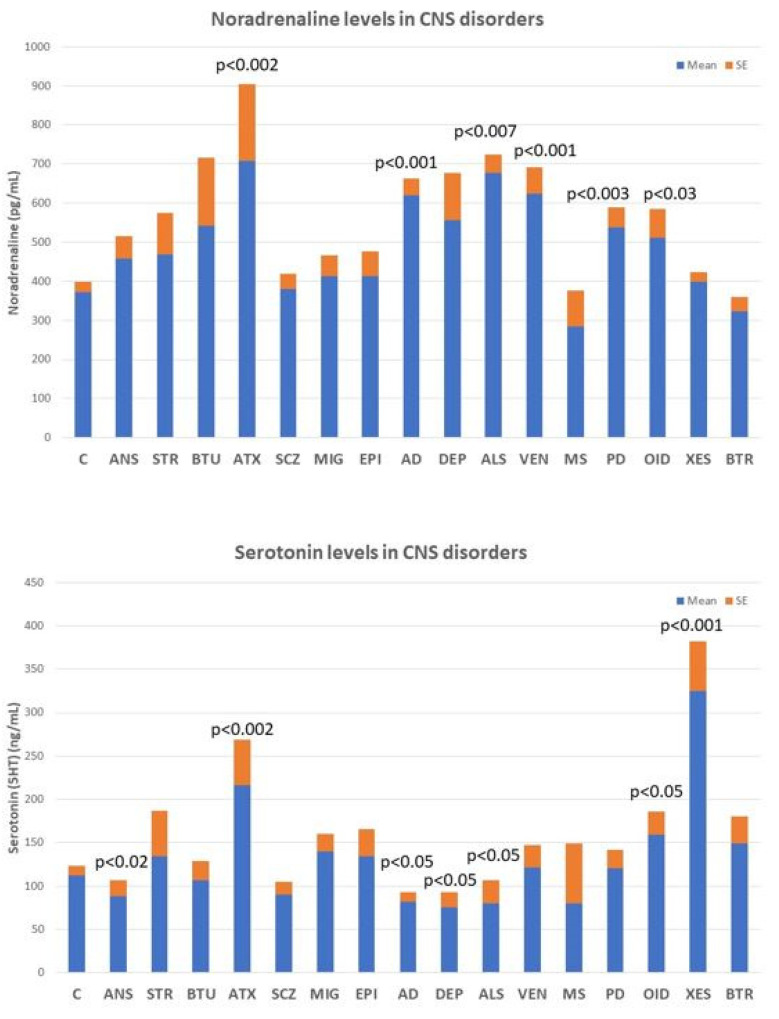
Noradrenaline (**upper panel**) and serotonin levels (**lower panel**) in central nervous system disorders. C: Control; ANS: Anxiety; STR: Stroke; BTU: Brain tumors; ATX: Ataxia; SCZ; Schizophrenia and psychosis; MIG: Migraine; EPI: Epilepsy; AD: Alzheimer’s disease; DEP: Depression; ALS: Amyotrophic Lateral Sclerosis; VEN: Vascular encephalopathy; MS: Multiple Sclerosis; PD: Parkinson’s disease; OID: Organic Intellectual Disability; XES: Xenoestrogenic syndrome; and BTR: Brain trauma.

Deficits in noradrenaline, dopamine, serotonin, histamine, GABA, glutamate, and various neuropeptides (GRF, CRF, somatostatin, and vasopressin) are particularly relevant, the alteration of which can lead to AD-related neuropsychiatric disorders. However, none of these biomarkers are sufficiently sensitive or specific to AD (Figure 5), although their quantification in CSF or blood is useful for monitoring brain damage and/or the efficacy or ineffectiveness of the treatments the patient receives. 

Noradrenaline levels in the blood increase significantly in most neurodegenerative diseases, including ataxic syndromes (*p* < 0.002), AD (*p* < 0.001), amyotrophic lateral sclerosis (ALS) (*p* < 0.007), and Parkinson’s disease (*p* < 0.003), as well as in vascular encephalopathies (*p* < 0.001) and in OID (Figure 5, upper panel). In contrast, serotonin levels tend to decrease in anxiety (*p* < 0.02), AD (*p* < 0.05), depression (*p* < 0.05), and ALS (*p* < 0.05), and show high levels in ataxic syndromes (*p* < 0.002), OID (*p* < 0.05), and in xenoestrogenic syndrome (*p* < 0.001), a novel clinical entity present in women with chronic use of contraceptives or hormone replacement therapy (HRT) (Figure 5, lower panel).

### 4.4. Aβ/Tau Levels

The most popular biomarkers for AD in body fluids (CSF, plasma) are the quantification of amyloid-β (Aβ42), total tau (T-tau), and phosphorylated tau (P-tau) in the CSF (AD CSF profile: decreased Aβ42 levels together with increased T-tau and P-tau levels). Other CSF markers (synaptotagmin, rab3a, SNAP-25, and neurogranin) are also altered [40,41]. However, the heterogeneity of AD and the inconvenience of having to perform a lumbar puncture to obtain CSF do not allow these biomarkers to reach high quotas of sensitivity and specificity, nor generalized use.

## 5. Concomitant Disorders and Phenotype-Modifying Treatments

Most patients with dementia (>90%) require multifactorial treatments for the management of concomitant disorders and/or neuropsychiatric symptoms associated with dementia. The chronic administration of drugs from different categories increases the risk of ADRs and DDIs [17,87,88,89].

The most frequent concomitant disorders in AD cases are the following: systolic hypertension (21%), diastolic hypertension (28%), obesity (>70%), diabetes mellitus type 2 (26%), hypercholesterolemia (40%), hypertriglyceridemia (20%), hyperuricemia (6%), metabolic syndrome (20%), transaminitis (11%), hyperbilirubinemia (15%), endocrine disorders (5%), iron deficiency anemia (7%), folate deficit (17%), vitamin B12 deficit (10%), cardiovascular disorder (40%), cerebrovascular disorder (>90% in patients over 80 years of age), anxiety (60%), depression (65%), behavioral disorders (20–90%), and cancer (10%). 

Cardiovascular risk factors (hypertension, hypercholesterolemia, and dyslipidemia) and ECG abnormalities are more frequent in males than in females. Hypertension is present in 21% of the cases. Systolic blood pressure is similar in females and males, but diastolic blood pressure is significantly higher in males than in females (*p <* 0.001) (Table 1). Cholesterol levels (Total, LDL) are higher in men and HDL-cholesterol and triglyceride levels are more elevated in females (Table 1). Brain damage and increased cognitive deterioration are currently associated with cardiovascular disorders and blood pressure changes in AD [90,91]. *APOE-4* carriers with dementia also exhibit cardiovascular disorders, atherosclerosis, and cerebrovascular damage [5,12,43,44,45,60,61,62,63,64,65,66,92]. Lipid metabolism disorders contribute to the cerebrovascular component of AD. Abnormalities in cholesterol metabolism and dysfunction of lipid rafts in cell membranes and arteriosclerosis are pathogenically relevant for cerebral ischemia and hypoperfusion, and accelerate premature neuronal death in patients who are predisposed to AD [44,93,94,95,96]. In contrast, the epidemiological link between diabetes and AD appears to be circumstantial, with no apparent pathogenic implications beyond the deleterious effects of hyperglycemia on brain function [97,98,99].

As a consequence of all these concomitant pathologies, patients with dementia consume a wide variety of drugs whose side-effects contribute to accelerating the degenerative process and cognitive decline. Of special importance are cardiovascular agents, statins, antidiabetics, antihypertensive drugs, analgesics, diuretics, bronchodilators, antirheumatics, and various categories of psychotropic drugs (neuroleptics, antidepressants, anxiolytics, hypnotics, and sedatives). The correct administration of these drugs requires a personalized therapeutic intervention, together with conventional anti-dementia treatments [15,17,100].

Combination treatments applied under pharmacogenetic guidance indicate that alterations in biochemical, hematological, and metabolic parameters affect drug efficacy and safety. Concerning cognitive function and neuropsychiatric disorders treated with multifactorial regimes, females and males respond differentially to treatment, showing a moderate improvement in cognition during the first year of treatment and significant improvements in anxiety and depression. 

Several pharmacogenetic studies agree that *APOE-4* carriers respond poorly to conventional treatments, while *APOE-3* carriers tend to respond better to different therapeutic regimens. Similarly, normal metabolizers (NMs) and intermediate metabolizers (IMs) associated with the different genotypes of the *CYP2D6*, *CYP2C9*, and *CYP2C19* genes show a better therapeutic response than poor metabolizers (PMs) and ultra-rapid metabolizers (UMs) to treatments with anti-dementia drugs and psychotropic drugs to regulate aberrant behaviors [24,60,61,62,63,64,65,66,67].

## 6. Alzheimer’s Disease Therapeutics and Drug Development

For the past 50 years, the major focus of pharmacological development in AD has been cognitive enhancers. The introduction of acetylcholinesterase inhibitors (AChEIs) in the early 1990s was the first option to restore cholinergic neurotransmission after the identification of a selective cholinergic deficit in the basal forebrain and neuronal loss in neocortex and hippocampus. Tacrine (9-amino-1,2,3,4-tetrahydroacridine) was the first AChEI introduced in 1993 for the treatment of AD. The Chinese product Huperzine A was approved in 1994. 

A new generation of AChEIs (donepezil, galantamine, and rivastigmine) was introduced years later. In 2003, the FDA approved memantine, an N-methyl-D-aspartate (NMDA) glutamate receptor partial inhibitor. Since then, no new FDA-approved drugs for AD were reported until the recent approval of the antibody aducanumab in 2021 [100,101].

The main categories of drugs studied during the last two decades as candidates for the treatment of AD were heterogeneous and include the following: neurotransmitter enhancers (11.38%), anti-amyloid agents (13.30%), anti-tau agents (2.03%), multi-target drugs (8.11%), novel synthetic drugs (8.13%), neuroprotective peptides (1.25%), old repository drugs (11.77%), anti-inflammatory drugs (1.20%), and a large number of natural products and derivatives (25.58%). Novel categories of therapeutic intervention (stem cell therapy, nanocarriers/nanotherapeutics) and combination treatments have also been challenged [101]. Over 2000 new AChEIs, some of them with dual inhibitory activity on acetylcholinesterase and butyrylcholinesterase, have been identified, as well as over 150 multi-target drugs [101,102]. Approximately 15% of pharmacological studies searching for anti-AD drugs focused on anti-amyloidogenic strategies (immunotherapy, APP modulators, α-secretase modulators, β-secretase (BACE) inhibitors, ϒ-secretase modulators, Aβ aggregation inhibitors, Notch inhibitors, β-sheet breakers, and Aβ scavengers), with notorious failures [101]. For the past two years, an increase in the number of disease-modifying agents targeting non-amyloid or tau pathogenic cascades has been observed [101,102,103,104].

### Immunotherapy

Since the early 2000s, several categories of vaccines and immunotherapeutic procedures have been developed for the treatment of AD, following the pioneering studies of Schenk and coworkers in 1999 [105]. For two decades, millions of dollars have been invested in passive and active immunotherapy in experimental AD models and in clinical trials. About 1000 papers on AD immunotherapy were published (85% anti-Aβ and 15% anti-tau) prior to the FDA approval of aducanumab as an immunotherapeutic strategy in mild-AD [103,106].

Active immunization studies demonstrate that presymptomatic immunization of PDAPP transgenic mice, overexpressing human mutant *APP* (Phe717Val), prevents Aβ-plaque formation, astrogliosis, and neuritic dystrophy. Immunization with Aβ42 reduces the extent and progression of AD-related neurodegeneration and improves cognition in the TgCRND8 murine model of AD [105,106,107,108]. Over 400 experimental studies with different immunization procedures were able to replicate these results. However, clinical studies did not show the same efficacy and initial clinical trials with the Aβ vaccine AN1792 had to be discontinued. This was because of severe undesirable effects, such as acute meningoencephalitis or microhemorrhagic lesions in the brain of some immunized patients. Some of these ADRs were attributed to a T-cell-mediated pro-inflammatory response; other mechanistic causes of brain damage in patients treated with active vaccines remain elusive. Improvements in immunization processes and vaccine preparations in recent years have made it possible to obviate many of these drawbacks and avoid adverse effects with the second-generation Aβ-active immunotherapies, anti-tau immunotherapies, and anti-Aβ monoclonal antibodies targeting Aβ epitopes [109,110,111,112,113,114]. Other modalities of immunization, such as dual vaccines (EB101) [111], Aβ3-10-KLH vaccine [112], or active full-length DNA-Aβ42 trimer immunization [113], also reduce amyloidogenic and tauopathic markers in transgenic animals [114].

On 7 June 2021, aducanumab was formally approved by the FDA as a putative disease-modifying antibody for the treatment of AD [115,116,117,118,119,120]. Aducanumab penetrates into the brain, binds parenchymal Aβ, selectively targets aggregated Aβ, and dose-dependently reduces Aβ levels, with parallel slowing of cognitive decline [116,117,118,119]. Vasogenic edema is one of the major ADRs of aducanumab and other injectable antibodies (BAN2401), especially in *APOE-4* carriers [120,121]. 

## 7. Pharmacogenomics

### 7.1. The Pharmacogenomic Machinery in Alzheimer’s Disease

The pharmacogenetic apparatus is made up of a genomic-epigenetic machinery composed of networks of genes that encode proteins, enzymes, and messengers involved in the therapeutic and/or toxic effects of each drug under the regulatory control of the epigenome [81]. 

The main pharmagenes encoding byproducts of the pharmacogenetic machinery involved in drug efficacy and safety can be classified into five major categories: pathogenic (Table 2, Table 3 and Table 4), mechanistic, metabolic, transporter, and pleiotropic genes [11,12,81]. Over 400 genes may influence drug pharmacogenetics; 240 genes have been associated with ADRs, and various rare variants have been identified in nearly 150 pharmagenes with clinical relevance [122,123].

**Figure 6:**
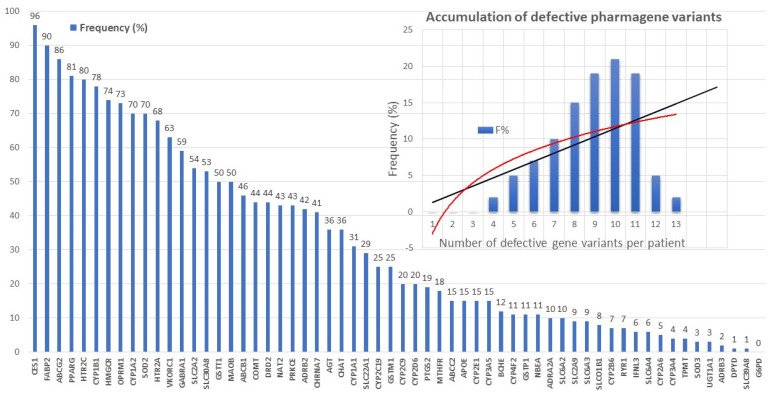
Frequency of defective pharmagene variants in Alzheimer’s disease and average accumulation of dysfunctional variants per patient.

Defective pharmagenes accumulate in approximately 85% of AD patients (Figure 6). The most frequent pharmagenes with dysfunctional effects in AD are the follows: *CES1* (96.45%), *FABP2* (90.86%), *ABCG2* (90.36%), *CYP1B1* (82.23%), *PPARG* (81.73%), *HTR2C* (79.19%), *HMGCR* (73.60%), *OPRM1* (69.54%), *SOD2* (67.51%), *VKORC1* (65.99%), *HTR1A* (65.48%), *SLCA2* (59.90%), *GABRA1* (55.33%), *GSTM1* (54.82%), *SLC30A8* (48.22%), *MAOB* (47.72%), *DRD2* (45.18%), *ABCB1* (44.16%), *NAT2* (43.65%), *AGT* (42.13%), *CHRNA7* (42.13%), *ADRB2* (41.62%), *PRKCE* (41.62%), *SLC22A1* (34.52%), *CHAT* (36.04%), *CYP1A1* (31.47%), *APOE* (30.96%), *COMT* (30.96%), and *GSTT1* (25.38%)(Figure 6).

Pharmagenes and their products are redundantly and promiscuously regulated by the epigenome (DNA methylation/demethylation, histone/chromatin remodeling, and miRNA regulation), thereby configuring the pharmacoepigenetic apparatus [81,124]. The same drug can affect the function of many different genes, and the same gene can influence the absorption, distribution, metabolism, and elimination (ADME) of various drugs [124].

### 7.2. Mechanistic Genes Involved in Cholinergic Neurotransmission

Mechanistic genes are those that encode proteins, enzymes and receptor subunits related to the mechanism of drug action. Key elements in cholinergic neurotransmission include ACh precursors (choline, acetyl-CoA), ACh synthesis (choline acetyltransferase), degradation enzymes (acetylcholinesterase, butyrylcholinesterase), choline transporter, vesicular ACh transporter, and cholinergic receptors (nicotinic, muscarinic).

Cholinergic neurons of the basal forebrain (basocortical cholinergic pathway, septohippocampal cholinergic pathway), where the nucleus basalis of Meynert is located, and cortical cholinergic projections, are the brain territories primarily affected in AD, with 60–80% depletion of cholinergic markers in severe cases [100].

Choline acetyltransferase (ChAT; EC 2.3.1.6), which catalyzes the biosynthesis of ACh from choline and acetyl-CoA, is encoded in the *ChAT* gene at 10q11.23. The first intron of the *ChAT* gene encodes the vesicular acetylcholine transporter (VAChT). VAChT transports ACh from the cytoplasm into the synaptic cleft. Mutations in *ChAT* and/or VAChT (*SLC18A3*) may represent potential susceptibility to AD [100].

Acetylcholinesterase (AChE; EC 3.1.1.7) is a serine hydrolase that hydrolyzes ACh to yield choline and acetate in the synaptic cleft; residual choline is recycled by the choline transporter and made available at the presynaptic level for de novo synthesis of ACh. AChE variants with catalytic activity include synaptic AChE (AChE-S) or tailed AChE (AChE-T), the most frequent variant in the brain; erythrocyte AChE (AChE-E) or hydrophobic AChE (AChE-H); and read-through AChE (AChE-R). The Yt erythrocyte blood group antigen system is inserted into the AChE molecule (AChE, His322Asn). A 4-bp deletion located 17 kb upstream of the transcription start site that abolishes 1 of 2 adjacent hepatocyte nuclear factor-3 (HNF3) binding sites causes hypersensitivity to AChEIs and severe CNS symptoms under low-dose exposure to pyridostigmine. AChE activity is decreased in AD brains, and APP is involved in the regulation of AChE [100,125].

Human serum cholinesterase (acylcholine acylhydrolase) or butyrylcholinesterase (BuChE) (EC 3.1.1.8) is a serine hydrolase that catalyzes the hydrolysis of ACh and choline esters such as the muscle relaxants succinylcholine and mivacurium. BuChE is a 574-amino acid protein encoded by the butyrylcholinesterase (*BCHE)* gene (4 exons, 64 kb) at 3q26.1. Over 30 genetic variants of *BCHE* have been described. In AD cases, the allelic frequency of the K-variant is >0.2 (vs. 0.09 in controls), and the risk for AD in carriers of the K-variant increases in the presence of the *APOE-4* allele in most studies. In mild cognitive impairment (MCI) cases, *BCHE-K* and *APOE-4* accelerate cognitive decline, hippocampal volumetric loss, and progression to AD [126].

The choline transporter (CHT, CHT1) is encoded by the *SLC5A7* (Solute Carrier Family 5 (Choline Transporter), Member 7) gene (2q12.3), with nine exons spanning 25 kb. CHT is a Na^+^- and Cl^−^-dependent high-affinity 580-amino acid protein (63.2 kD) with 12 transmembrane (TM) domains responsible for the uptake of choline for ACh synthesis in cholinergic neurons. An A-to-G transition at nucleotide 265, resulting in an Ile89-to-Val substitution (I89V) within the third TM domain, reduces the maximum rate of choline uptake by about 40%. The high-affinity choline transporter (CHT1, SLC5A7) expressed in cholinergic neurons represents a rate-limiting step for ACh synthesis. Alterations in CHT function decrease choline uptake and ACh synthesis, with a consequent impairment in cholinergic neurotransmission. CHT1 dysfunction may contribute to AD pathology. Aβ decreases choline uptake activity and cell surface CHT protein levels. CHT trafficking is different in wild-type *APP* (*APPwt*) and in Swedish mutant *APP* (*APPSwe*) SH-SY5Y human neuroblastoma cells. APP-CHT interaction is decreased in *APPSwe* transgenic mice [127,128].

Mutations in *PSEN1* may regulate cholinergic signaling via CHT1. Cortical neurons express active CHT1, and CHT1-mediated choline uptake activity is reduced in *PSEN1* M146V mutant knock-in mice [128]. In a mouse model of scopolamine-induced amnesia, scopolamine decreases ChAT, CHT, vesicular ACh transporter (VAChT), and muscarinic ACh receptor M1 (M1R) in the septum and hippocampus [100].

The vesicular acetylcholine transporter (VAChT) is encoded by the *SLC18A3* (Solute Carrier Family 18 (Vesicular Actylcholine), Member 3) gene (10q11.23). This gene encodes a transmembrane protein that transports ACh into presynaptic secretory vesicles to be released at cholinergic terminals in the CNS and peripheral nervous system. The *SLC18A3* gene is located within the first intron of the *ChAT* gene. Mutations in the *SLC18A3* gene cause presynaptic congenital myasthenic syndrome-21 (CMS21). Nitrosylation of VAChT is increased in the frontal cortex and hippocampus of *APP/PS1* mice [129]. *B6.eGFPChAT* congenic mice with multiple gene copies of *VAChT* exhibit high VAChT protein expression in the hippocampal formation, accompanied by enhanced ACh release [130]. Mice with a targeted mutation in the *SLC18A3* gene show a 40% reduction in transporter expression, with memory deficits that can be reversed with AChEIs. Diminished expression of the splicing regulator hnRNPA2/B1BACE1 causes abnormal splicing in *BACE1*; increased APP processing; accumulation of Aβ; and increase in GSK3, tau hyperphosphorylation, caspase-3 and neuronal death. In human brain, there is a correlation between decreased levels of VAChT and hnRNPA2/B1 levels and increased tau hyperphosphorylation [131]. There is a selective loss of cholinergic terminals in the neocortex and hippocampus of double transgenic (*APP-K670N/M671L* + *PS1-M146L*) mice, with relevant alterations in VAChT. The levels of ChAT, AChE, and BuChE are similar in the hippocampus of young *apoE4* and *apoE3* mice. ChAT levels decrease more in *apoE4* than in *apoE3* mice. The levels of muscarinic receptors are also higher in *apoE4* mice. ACh release from hippocampal slices is reduced in old *apoE4* mice in parallel with reduced VAChT levels [132].

The distribution of the vesicular acetylcholine transporter in early AD shows a decrease of about 47–62% in the cingulate cortex and parahippocampal-amygdaloid complex. The numbers of ChAT and VAChT neurons correlate to the severity of dementia and show no relationship with APOE status. Cholinergic baso-cortical and septo-hippocampal pathways are particularly damaged in AD, as reflected by PET studies of the VAChT [133].

Nicotinic and muscarinic ACh receptors are the final effectors of cholinergic neurotransmission. Nicotinic receptors (nAChRs) play an important role in the prefrontal cortex, where cortical and subcortical inputs are integrated to execute higher activities of the CNS (learning, attention, working memory planning, decision-making, and perception of reality). Mutations in the *CHRNB2* or *CHRNA7* genes that encode the nicotinic receptor β2 and α7 subunits can lead to brain disorders, including AD [134]. α7nAChR, encoded by the *CHRNA7* gene, is involved in AD pathogenesis connected to hypocholinergic neurotransmission and Aβ deposition. Carriers of the *CHRNA7* rs7179008 variant showed decreased risk of dementia. Single-nucleotide polymorphisms (SNPs) in the *CHRNA7* or *CHRFAM7A* genes may affect susceptibility to AD. *CHRFAM7A*-2-bp deletion or *CHRNA7* SNPs (rs1514246, rs2337506, and rs8027814) might be protective for AD [100,135].

Loss of basal forebrain cholinergic neurons correlates with cognitive decline in AD. Exposure to Aβ up-regulates neuronal α7nAChRs and increases neuronal excitability, and α7-nAChRs mediate, in part, Aβ-induced neurotoxicity, which is prevented by either the α7-nAChR antagonist methyllycaconitine or by α7 subunit gene deletion. In contrast, it appears that α7nAChR selective agonists (e.g., PHA-543613) and galantamine may ameliorate Aβ-impaired working and reference memory, suggesting that α7 nAChR activation reduces Aβ-induced cognitive deficits, whereas receptor blockage increases Aβ toxicity and cognitive impairment [136].

### 7.3. Metabolic Genes

Metabolic genes encode Phase-I-II reaction enzymes in the liver and other tissues. Phase-I reaction enzymes include cytochrome P450 family (CYPs) of mono-oxygenases; alcohol, aldehyde, dihydropyrimidine, and short-chain and xanthine dehydrogenases; carbonyl, aldo-keto, glutathione, and cytochrome b5 reductases; peptidases; cytidine deaminases; amine oxidases; esterases; prostaglandin endoperoxide synthases; epoxidases; flavin-containing monooxygenases; and superoxide dismutases. Phase-II reaction enzymes include glucuronosyl transferases, methyl transferases, amino acid transferases, glutathione transferases, N-acetyl transferases, dehydrogenases, esterases, thioltransferase, and sulfotransferases [4,5,6,9].

Most PGx studies with AChEIs in AD are related to CYPs. Nearly 30% of AD cases are deficient for CYP2D6 and CYP3A4/5 enzymes associated with the metabolism of AChEIs. About 80% of AD patients in the Caucasian population are deficient metabolizers for the tetragenic *CYP2D6*, *2C19*, *2C9*, and *3A4/5* cluster. CYP2D6, 2C19, 2C9, and 3A4/5 enzymes metabolize 60–80% of current drugs. Most metabolic enzymes show ontogenic-, age-, sex-, circadian-, and ethnic-related differences. In terms of distribution and frequency of CYP geno-phenotypes (Normal Metabolizer: NM; Intermediate Metabolizer: IM; Poor Metabolizer: PM; and Ultra-Rapid Metabolizer: UM), there is a great geographic and ethnic variability worldwide [11,12,81].

### 7.4. Transporter Genes

There are over 200 transporter proteins responsible for the transfer of endogenous and exogenous substances through the blood–brain barrier. The main categories of transporters, with some relevance in AD, include ATPase (P-type, V-type, and F-type subfamilies), ATP-binding cassette transporters (Subfamilies ABC1, MDR/TAP, CFTR/MRP, ALD, OABP, GCN20, and WHITE), and Solute carriers (high-affinity glutamate and neutral amino acid transporter family) (SLC) [81]. ABC and SLC variants are involved in the pathogenesis of dementia, and a large number of ABC transporters influence the efficacy and safety of more than 1000 different drugs, including AChEIs and memantine [11,12,81].

Many other transporters are associated with AD pathogenesis [100]. Defective transporters are attractive candidates for therapeutic intervention in AD, and their mutations should be considered in PGx strategies [100,137].

### 7.5. Pharmacogenetics of Acetylcholinesterase Inhibitors

#### 7.5.1. Donepezil

Donepezil is the second AChEI approved for the treatment of AD, after Tacrine, and the most prescribed drug for dementia worldwide [69,138,139,140,141] (Table 4). Donepezil is a selective AChEI with a long elimination half-life (t_1/2_) of 70 h and is metabolized in the liver [141,142,143]. Donepezil clearance is 7.3 l h^−1^ with gender- and inter-individual variability (30%) [141]. Donepezil increases brain ACh levels by 35% and decreases AChE activity by 40–90%, with no effect on ChAT, vesicular ACh transporter, CHT, or muscarinic receptors. Donepezil might also exert some beneficial effects against Aβ-induced neurotoxicity. Donepezil inhibits AChE and BCHE; is a major substrate of CYP2D6, CYP3A4, AChE, and UGTs; and is transported by ABCB1 [141,142,143] (Table 4). Individual variation in metabolic genes (*CYP2D6*) and pathogenic genes (*APOE*) modulates the response to donepezil treatment [139,141,142,143,144]. Both *APOE* and *CYP2D6* variants are determinant for donepezil efficacy and safety in AD patients [139,141,142,143,144].

*APOE-3* carriers are the best responders, and *APOE-4* carriers are the worst responders to donepezil in either monotherapy or in drug combination regimes; *CYP2D6*-EMs tend to be good responders, and *CYP2D6*-PMs exhibit a poorer response to donepezil, with probably more ADRs [11,12,66,67,138,139,143,144].

ABCA1 regulates cholesterol transport and APOE metabolism. AD patients with the *ABCA1* rs2230806 G/G genotype respond better to donepezil than carriers of the *A/A* and *A/G* genotypes, and *ABCA1* rs2230806 G/G-*APOE3* non-carriers show a better clinical response to donepezil. Patients homozygous for the *T/T/T* genotype in the *ABCB1* haplotypes *1236C*/*2677G*/*3435C* and *1236T*/*2677T*/*3435T* show lower plasma donepezil concentration-to-dose ratios and better clinical response to donepezil. Donepezil may inhibit ABCB1 [100].

Patients harboring the APOE-ɛ4/BCHE-K* genotype show an earlier age of onset, an accelerated cognitive decline, and an irregular response to donepezil. In patients with MCI, donepezil accelerates cognitive decline in homozygous *BCHE-K* and *APOE-4* carriers. The *BCHE-K* variant is associated with lower AChE hydrolyzing activity, and BuChE activity increases in parallel with disease progression. Donepezil treatment is not recommended in *BCHE-K* and *APOE-4* carriers with MCI or dementia [145].

Donepezil may induce upregulation of α7nAChR protein levels, potentially protecting neurons against neurodegeneration. *CHRNA7* rs8024987 (C/G) and rs6494223 (C/T) respond better to donepezil. Donepezil-induced α7nAChR upregulation is higher in *T/T* carriers (7–15%) than in *C/C* or *C/T* carriers [146].

Donepezil treatment tends to increase APP forms in *APOE-4* non-carriers and interacts with many drugs, causing cardiotoxicity [100].

#### 7.5.2. Galantamine

Galantamine is a reversible AChEI with allosteric modulatory effects on nicotinic ACh receptors. This drug is rapidly absorbed (C_max_ = 1 hr), with low protein binding (28.3–33.8%), a steady-state volume of distribution (V_ss_) of 193 L, and an elimination half-life of 7–8 h (20–25% is excreted unchanged in urine) [147]. Median clearance in female/male AD patients is 12.4/14.8 L/h, probably due to body weight differences rather than a real gender effect. In patients with liver dysfunction, the metabolic clearance is reduced by 60%. Galantamine increases the levels of brain VAChT. Galantamine acts as a major substrate of CYP2D6, CYP3A4, ABCB1 and UGT1A1, and as an inhibitor of AChE and BCHE. Galantamine efficacy and safety can be modified by *APOE*, *APP*, *AChE*, *BCHE*, *CHRNA4*, *CHRNA7*, and *CHRNB2* variants [100,141,147] (Table 4). CYP2D6 and CYP3A4 enzymes are responsible for galantamine metabolism. Glucuronidation, N-demethylation, O-demethylation, epimerization, and N-oxidation are its major metabolic pathways. Galantamine pharmacokinetic parameters mainly depend on *CYP2D6* variants. *CYP2D6*-PMs exhibit 45–61% higher dose-adjusted galantamine plasma concentrations than *CYP2D*6-NMs and IMs [141,147], without substantial effects on pharmacodynamics. There is no linear correlation between galantamine concentration and cognitive response in AD patients. Interaction with foods may modify the effects and bioavailability of galantamine [100].

Several studies indicate that galantamine in AD may show better results in *APOE-4* non-carriers. Patients with MCI treated with galantamine for one year also showed a lower rate of whole brain atrophy, preferentially among *APOEϵ4* carriers. Others suggest no major influence of *APOE* variants in the effects of galantamine in AD. *CHRNA7* rs8024987 variants may also affect galantamine in females [100].

#### 7.5.3. Rivastigmine

Rivastigmine (ENA 713, carbamoylatine) is a dual AChEI with brain-region selectivity (>40% AChE inhibition) and a long-lasting effect. Rivastigmine also inhibits peripheral BuChE (>10%).

Saturable first-pass metabolism leads to 35% bioavailability of the administered dose and nonlinear short half-life pharmacokinetics, with renal elimination. Rivastigmine is a pseudo-irreversible dual inhibitor of AChE and BCHE with a very short t_1/2_ (1–2 h) and longer duration of action due to blockade of AChE and BCHE for around 8.5 and 3.5 h, respectively.

Rivastigmine increases VAChT and ChAT expression in the frontal cortex, hippocampus, striatum, and cerebellum, providing additional effects on cholinergic neurotransmission. Liver and intestine esterases metabolize rivastigmine [141,143,148], whereas *ChAT*, *AChE*, *BCHE*, *CHRNA4*, *CHRNB2*, *APOE*, *APP*, and *MAPT* variants may affect rivastigmine pharmacokinetics and pharmacodynamics (Table 4). Rivastigmine is more effective in *APOE-4* non-carriers in different ethnic groups. CYP enzymes are not involved in the metabolism of rivastigmine. *UGT2B7*-PMs show a deficient response to treatment and higher rivastigmine levels. CYP2D6*3, *UGT2B7*, and UGT1A9*5 carriers, in combination treatments with memantine, show differential responses to treatment. *CHRNA7* variants and two intronic SNPs of *ChAT* (rs2177370 and rs3793790) influence the response to AChEIs. Lower response to rivastigmine and reduced enzyme activity are observed in patients harboring the *BCHE-K*-variant (rs1803274). SNPs outside the coding sequence of the *BCHE* gene (rs1126680, rs55781031) and the K-variant (p.A539T) may reduce enzyme activity. Carriers of these deleterious SNPs should receive lower doses of rivastigmine or start treatment with a different AChEI. *BCHE-wt/wt* females show a better response to rivastigmine than males. Males with the BCHE-K* variant tend to show faster cognitive decline than females. Rivastigmine may attenuate the progression of cognitive decline in male *BuChE-K* and in female *BuChE wt/wt*. Patients with the bigenic *BCHE-K-APOE-4* genotype are poor responders to rivastigmine patch or memantine add-on therapy [100].

#### 7.5.4. Huperzine A

Huperzine A is a natural *Lycopodium* sesquiterpene alkaloid extracted from the Chinese medicinal plant *Huperzia serrata*. This compound is a reversible and highly selective second-generation AChEI for AD and was approved in China in 1994 [149]. Huperzine A pharmacokinetics show age-dependent differential features. Age is a covariate with influence on huperzine A clearance. The plasma concentration-time profile of huperzine A in elderly patients reveals a one-compartment model with first-order absorption and elimination.

CYP1A2 is the major metabolizing enzyme of huperzine A in rat liver microsomes; secondary metabolizing enzymes are CYP3A1/2, CYP2C11, and CYP2E1. In humans, huperzine A is excreted unchanged by kidney, with no apparent involvement of CYP1A2, CYP2A6, CYP2C9, CYP2C19, CYP2D6, CYP2E1, or CYP3A4 enzymes [150] (Table 4). At a toxicological dose in rats, huperzine A may induce CYP1A2 by enhancement of transcription. Carboxylesterases (CESs) (CES1 and CES2) are enzymes catalyzing the hydrolysis of ester, amide, and carbamate chemicals. CESs might be tangentially involved in huperzine A metabolism.

Huperzine A penetrates the brain and interacts with ABCB1 and ABCG2 efflux transporters. Huperzine A is a substrate of ABCB1. In *Abcb1a−/−* mice, the brain to plasma concentration ratio of huperzine A is higher than in wild-type animals [100].

### 7.6. Pharmacogenetics of Memantine

Memantine is a non-competitive low-affinity NMDA receptor antagonist. Its long t_1/2_ is about 70 h, and it is eliminated unchanged via the kidneys; however, several genes can influence its efficacy and safety [141]. Memantine displays its therapeutic effect by inhibition of glutamate via NMDA receptors, with partial antagonistic activity on GRIN2A, GRIN2B, GRIN3A, HTR3A, and CHRFAM7A. Memantine efficacy and safety may be modulated by pathogenic genes (*APOE, PSEN1*, and *MAPT*) and mechanistic genes (*GRIN2A*, *GRIN2B*, *GRIN3A*, *HTR3A*, *CHRFAM7A*, *c-Fos*, *Homer1b*, and *PSD-95*). Memantine is a strong inhibitor of CYP2B6 and CYP2D6 and a weak inhibitor of CYP1A2, CYP2A6, CYP2C9, CYP2C19, CYP2E1, and CYP3A4 [141,143,151]. In human liver microsomes, memantine inhibits CYP2B6 and CYP2D6; decreases CYP2A6 and CYP2C19; and has no effect on CYP1A2, CYP2E1, CYP2C9, or CYP3A4. When co-administered with CYP2B6 substrates, the metabolism of memantine is decreased by 65%. Memantine clearance is highly dependent on the *NR1I2* rs1523130 variant. *NR1I2* rs1523130 CC carriers show a faster elimination rate than *CT* or *TT* carriers [151].

Memantine transport across the blood–brain barrier (BBB) might be facilitated by proton-coupled organic cation antiporters. In AD patients, memantine can be used as a monotherapy or in combination with AChEIs. Memantine induces proteomic changes in the hippocampus and the cerebral cortex of transgenic mice (3 × Tg-AD), with modifications in the expression of 233 and 342 proteins, respectively. Memantine also reduces cerebrovascular Aβ and hemosiderin deposits in *APP23* transgenic mice with cerebral amyloid angiopathy, by enhancing Aβ-cleaving insulin degrading enzyme (IDE) expression. Memantine increases histamine neuron activity, as reflected by a 60% increase in brain tele-methylhistamine levels and an increase in hypothalamic H3 autoreceptors, where histamine neurons are located [100].

### 7.7. Pharmacogenetics of Aducanumab

Up to now, no reliable information has been available on the pharmacogenetics of aducanumab and no specific studies have been reported in this regard. Based on its potential mechanism of action, as a scavenger of Aβ, it can be inferred that different mutations in the *APP*, *PSEN1*, and *PSEN2* genes, related to the intensity of the Aβ load in neuritic plaques, affect the efficacy of this monoclonal antibody. It also seems clear that the *APOE* genotype affects the safety and efficacy of aducanumab. *APOE-4/4* carriers not only respond poorly to aducanumab but worsen and develop severe side-effects, neuroinflammatory reactions, perivascular microedema, and white matter lesions [118,119,120].

Chronic administration of aducanumab to *tgAPPPS1-21* mice modifies the expression of a series of proteins associated with AβPP trafficking/processing, neuronal cytoskeleton, stress response, metabolism, and mitochondria. If some of these pathways and their respective proteins, potentially implicated in AD pathogenesis, are affected in humans, it is likely that mutations in the corresponding genes might affect aducanumab efficacy and safety [152].

By analogy with other monoclonal antibodies, such as Abciximab, a Fab antibody fragment of the chimeric human-murine monoclonal antibody 7E3 that inhibits platelet aggregation by binding to IIb/IIIa receptors, or Trastuzumab, an immunoglobulin G1 (human-mouse monoclonal rhuMab HER2γ1-chain antihuman p185c-erbB2 receptor), which binds to the extracellular domain of human epidermal growth factor receptor 2 protein (HER-2) in breast neoplastic cells overexpressing HER-2, it cannot be excluded that aducanumab might be processed via phase-I enzymes of the CYP family, phase II enzymes, and CNS transporters (ABC and SLC families), or that mutagenic variants that affect genes responsible for neuroimmune cascades (*IL1B*, *IL6*, *TNF*) might modify the therapeutic response and toxicity of aducanumab [143].

### 7.8. Pharmacogenetics of Multifactorial Treatments

Multifactorial treatments should be designed in a personalized fashion with neuroprotective drugs, anti-dementia drugs, drugs to control concomitant pathologies, psychotropic drugs for the treatment of neuropsychiatric disorders, and other medications especially important for specific metabolic deficits. In these cases, the use of pharmacogenetic procedures is particularly useful, as illustrated in the following sections.

Most studies with multifactorial combination therapies have used the *APOE* gene and *CYP* gene variants as reference genes in pharmacogenetics. The main conclusions obtained from these studies agree that the best responders to conventional treatments with AChEIs, memantine, and various neuroprotective agents are patients with the *APOE-3/3* genotype, while the worst responders are patients with the *APOE-4/4* genotype. Intermediate responses are observed in patients with *APOE-2/4* and *APOE-3/4* genotypes, where the presence of the *APOE-4* allele gives them the character of not good responders.

When analyzing the pharmacogenetics of multifactorial treatments based on CYP variants (*CYP2D6*, *CYP2C9*, *CYP2C19*, and *CYP3A4/5*), in most cases, in Caucasian patients (with large variations in other ethnic groups), it is observed that carriers of *CYP2D6*-NM geno-phenotypes are the best responders, while *CYP2D6*-PMs tend to be poor responders to common treatments. Carriers of *CYP2D6*-IM and *CYP2D6*-UM geno-phenotypes show intermediate responses between those observed in NMs and PMs [11,12,13,15,24,44,60,66,67,100].

Since the *APOE* and *TOMM40* genes occupy adjacent loci, it has been shown that both genes influence the pathogenesis of AD and the therapeutic response to anti-dementia treatments. In the first pharmacogenetic study in AD with the bigenic genotype *APOE-TOMM40*, carriers of the *TOMM40 poly T-S/S* genotype were good responders, similar to *APOE-3/3* carriers, while patients harboring the *L/L* variant were the worst responders. Carriers of *VL/VL* and *S/VL* variants showed an intermediate response to multifactorial treatments. When analyzing the effect of both genes on the therapeutic response in AD patients, carriers of the *APOE-4/4* genotype in haplotypes with *TOMM40-L/L* and *S/L* variants responded the worst to treatment. In contrast, carriers of the *APOE-3/3* genotype in haplotypes with the *TOMM40-S/S* variant, and to a lesser extent *TOMM40-S/VL* and *TOMM40-VL/VL* variants, were the best responders to anti-dementia treatments. The *APOE-4/4* genotype is exclusively associated with the *TOMM40-L/L* genotype in 100% of AD cases. It has been suggested that this haplotype (*APOE-4/4-TOMM40-L/L*) is responsible for premature neuronal death, early onset of the disease, accelerated cognitive decline, and a poor response to conventional treatments [22].

### 7.9. Pharmacoepigenetics

Various epigenetic aberrations, present in AD patients, contribute to enhancing brain neurodegeneration through the abnormal expression of pathogenic genes. Similar epigenetic alterations can alter the expression and function of the mechanistic, metabolic, transporter, and pleiotropic genes that constitute the pharmacoepigenetic apparatus, conditioning the response to drugs [81,82,100].

Very few pharmacoepigenetic studies have been carried out in AD, and there are no epigenetic drugs capable of protecting neurons against the process of premature death to which they are subjected in AD. Among the many epigenetic factors that influence the pathogenesis of AD and the response to drugs, different variants in the genes that encode sirtuins could alter the epigenetic machinery with deleterious effects on the brain. For example, the *SIRT2-C/T* variant (rs10410544) increases vulnerability to AD in *APOEε4*-negative patients. In the first pharmacoepigenetic study with the bigenic cluster *SIRT2-APOE*, 18 haplotypes were identified with potential influence on the therapeutic response to multifactorial regimens with anti-dementia drugs. The *APOE-4* allele accumulates in *SIRT2-T/T* and *SIRT2-C/T* carriers. *SIRT2-C/C* carriers are the worst responders, *SIRT2-C/T* carriers are the best responders, and *SIRT2-T/T* carriers are intermediate responders to combination treatments. When studying the therapeutic response by bigenic clusters of *APOE-SIRT2*, it was found that patients with the *33CC* genotype were the ones who responded best to treatment; *33TT* and *34CT* carriers showed an intermediate response, and *24CC* and *44CC* carriers showed the worst response to multifactorial treatment. A similar analysis of the bigenic cluster *SIRT2-CYP2D6* showed that the best responders were patients carrying the geno-phenotype *SIRT2-C/T-CYP2D6*-EM [13].

### 7.10. Pharmacogenomics of Mood Disorders and Anxiety

The treatment of depression and anxiety in cases of dementia is always inserted into multifactorial therapeutic regimens with anti-dementia agents and complementary treatments for the control of concomitant pathologies. The addition of antidepressants and anxiolytics in small doses to these therapeutic regimens is usually well tolerated. In personalized protocols, a similar favorable response is observed in men and women. *APOE-4* carriers tend to respond worse to antidepressants and anxiolytics than *APOE-3* carriers. Patients with the geno-phenotypes *CYP2D6*-NM, *CYP2C9*-NM, and *CYP2C19*-NM usually respond better than PMs or UMs; IM patients show an intermediate response to antidepressants. In contrast, patients with mutations in the *CYP3A4* and *CYP3A5* genes show an abnormal response to benzodiazepines, when compared to *CYP3A4/5*-NMs. *CYP3A4/5*-PMs show a more pronounced effect with an excess of side-effects to benzodiazepines, and *CYP3A4/5*-UMs respond poorly to benzodiazepines due to ultra-rapid metabolization of the drugs and the consequent lack of therapeutic effect [24,67,153,154,155].

In patients with depression treated with antidepressants chosen by trial and error, according to the pharmacological experience of the prescribing physician, the rate of error and therapeutic failure is over 50%. When these patients receive the right drug, in correct doses, adapted to their pharmacogenetic profile, an improved therapeutic response is obtained in over 60% of cases between one and three months of personalized treatment [156].

## 8. Future Trends

Apart from the standard care needed by every patient with dementia, in the overall management of AD it is important to improve diagnostic accuracy (>30% error), correctly identify and treat concomitant pathologies, implement presymptomatic diagnostic protocols, personalize pharmacological treatments, and initiate prevention programs in the population at risk. A major problem in the management of AD is the lack of curative treatments; polypharmacy related to concomitant pathologies; and unnecessary abuse of psychotropic drugs, especially in nursing homes.

Once the differential diagnosis of AD has been established, with diverse procedures [40,41,42,46,157,158,159,160,161], it is necessary to optimize the therapeutic resources adapted to each form of dementia, depending on the predominance of cognitive and behavioral impairment over the motor component or vice versa [162].

In people over 80 years of age, practically >95% of patients consume more than ten different medications per day for the treatment of ailments that contribute to the aggravation of the clinical picture of dementia (cardiovascular diseases and associated pathologies, cerebrovascular insufficiency and cerebral microinfarctions, metabolic disorders, arthropathies, etc.) [163,164,165,166,167,168,169].

When polypharmacy is unavoidable, pharmacogenetic procedures should be implemented for the personalization of indispensable pharmacological treatments to avoid ADRs and DDIs [17]. In the Caucasian population, only 20% of the cases are extensive metabolizers for drugs metabolized via CYP2D6-2C9-2C19-3A4/5 enzymes. This implies that by trial and error, the treatments administered to these patients, without knowledge of their pharmacogenetic profile, in more than 60% of cases will harm or will not provide any benefit, from a therapeutic point of view [12,14,15,17,23,100].

In addition to the conventional treatments available for different forms of dementia, assuming their limitations in terms of the cost–benefit ratio, and without forgetting that the search for etiopathogenic treatments is an unavoidable priority, the search for new forms of symptomatic treatments today should not be ruled out [169], as well as the use of alternative therapies that contribute to alleviate dysfunction, disconnection from the socio-family environment, and aberrant behaviors present in a high number of patients with dementia [170,171,172,173,174]. Furthermore, a deeper understanding of AD pathophysiology may lead to the identification of neuronal signaling pathways as potential novel targets for therapeutic intervention (i.e., tyrosine kinases) [175,176,177,178,179].

Once the necessary treatments have been established, therapeutic follow-up programs with reliable biomarkers should be implemented to guarantee the efficacy of the treatments administered and to eliminate those ineffective drugs that could also contribute to aggravating the clinical picture of dementia.

In terms of prevention, genomic screening programs should be implemented to identify the population at risk. These programs should include genomic clusters with the most informative pathogenic genes at a reasonable price. New NGS and GWAS techniques must be validated across different ethnicities to effectively cover genomic risk.

There are currently over 4000 human genes for which no functional or structural assignment has been established nor an alteration (either structural -SNP- or functional -epigenetic-related abnormal gene expression) associated with any pathology. It is expected that in the coming years, some of these genes will show subtle variants potentially associated with rare diseases and/or degenerative processes. On the other hand, Figure 3 and Figure 6 clearly illustrate the accumulation of various genetic variants in the same patient, supporting a possible universal rule in genomics. A common rule to all complex, polygenic, and multifactorial diseases, such as AD is that the greater the number of genes affected, the earlier the onset of the disease, the more accelerated its clinical course and the worse the response to conventional treatments; the fewer genes affected, the later the onset of the disease, the slower the clinical course, and the more favorable therapeutic response.

An important issue is to validate the weight that each polymorphic variant has in the pathogenesis of AD. APOE is a paradigmatic example. Everyone accepts that the *APOE-4* allele is a major risk factor for AD. However, technical difficulties, lack of interest (or both) mean that the pathogenic role of *APOE-4* has been negligently considered until now in terms of therapeutics [101,180].

It is highly unlikely that current treatments, including aducanumab, will contribute to the efficient management of therapeutic deficiencies of AD. Under optimal conditions (and only in presymptomatic cases or in very mild cases), aducanumab may be useful in less than 20% of patients, without forgetting that in some cases (i.e., *APOE-4* carriers), its effects could be deleterious [115,116,117,118,119,120,121].

Another drawback is the high cost of treatment, which will hardly be borne by the public health system in many countries and will establish greater inequality between rich and poor.

The only way to effectively fight AD is through prevention. AD is destroying the brains of the at-risk population since the brain stops maturing at around 30–35 years of age. From then, it can take more than 30 years before symptoms appear. That is the time available to the patient and the medical community to intercept the course of the disease and slow or stop the neurodegenerative process in genomically-vulnerable individuals. For this, it is necessary to implement preventive programs with prophylactic interventions and treatments aimed at protecting the brain against the destructive process of neurodegeneration. Among the preventive strategies, all the intercurrent diseases, which anyone throughout life can suffer from and whose inadequate treatment can contribute to neuronal damage, should not be ignored.

At present, no preventive strategy is specific enough to effectively combat premature neurodegeneration associated with AD because, among other things, the etiopathogenesis of the disease itself is in question as it focuses primarily on the pathogenic binomial of amyloidosis-tauopathy with little therapeutic success over two decades [181,182,183,184]. However, any future preventive strategy should be based on the following methodological steps: (i) identification of the population at risk by global genomic screening; (ii) identification of potential concomitant pathologies that increase brain risk; (iii) initiation of multifactorial prophylactic intervention once the brain stops maturing; (iv) monitoring of the efficacy of the preventive strategy with epigenetic, proteomic, and metabolomic biomarkers; and (v) prophylactic intervention of concomitant pathologies and/or treatment of symptomatic ailments.

The practical application of pharmacogenetics based on the individual genomic profile of each patient requires the following: (i) adjusting the dose of each treatment according to the condition of NM (regular dose), IM (50% reduction of the usual dose), PM (avoid drugs whose only metabolization route is associated with the mutant enzyme), or UM (increase the dose by 25%; if there is no response or side effects appear, change the drug regime); (ii) avoid co-administration of drugs that are substrates and inhibitors of the same metabolic enzyme; (iii) in IMs, use the help of inducers that enhance the metabolic capacity of the enzyme with decreased activity; (iv) in some cases, it would be possible to modulate the enzymatic activity of mutant genes with epigenetic drugs; and (v) avoid any treatment for which there is evidence of toxicity from the patient’s mutant condition (12–15, 17, 22–24).

When pharmacogenetics become a mature discipline, the most effective manner to optimize the available therapeutic resources and reduce ADRs and DDIs is the implementation of pharmacogenetic procedures as a daily routine in the clinic. The routine use of pharmacogenetics is limited by a series of educational factors (information deficit among health professionals), technical factors (poor characterization of the pharmacogenetic properties of more than 50% of commonly used drugs), biomedical factors (shortage of biomarkers of drug efficacy and toxicity), economic factors (high cost of pharmacogenetic screening), administrative factors (lack of organization in hospitals and health centers for personalized medicine), and regulatory factors (poor definition of pharmacogenetics parameters by regulatory agencies for the use of drugs and for the development of new pharmaceutical products) [185,186].

## 9. Conclusions

Men and women show substantial differences in their AD-related phenotypes, including anthropometric, biochemical, hematological, metabolic, hormonal, neurotransmitter, cardiovascular, and cerebrovascular parameters, as well as cognition, mood, behavior, genomic, and pharmacogenomic profiles. The most frequent concomitant diseases in AD patients are hypertension (>25%), obesity (>70%), diabetes mellitus type 2 (>25%), hypercholesterolemia (40%), hypertriglyceridemia (20%), metabolic syndrome (20%), hepatobiliary disorder (15%), endocrine/metabolic disorders (>20%), cardiovascular disorder (40%), cerebrovascular disorder (60–90%), neuropsychiatric disorders (60–90%), and cancer (10%).

Cognitive markers indicate than females show worse cognitive performance than males. Likewise, depression and anxiety are also more prevalent in women than in men. The ECG is abnormal in 40% of the patients (38% F; 43% M). No sex-related differences are found in MRI anomalies, which are present in over 70% of the cases.

In addition to conventional clinical markers, the most useful biomarkers for a predictive diagnosis or diagnostic confirmation of antemortem AD are genomic markers, epigenetic biomarkers, neurotransmitters, and levels of Aβ/tau in the brain (PET Scan) and/or in body fluids.

The genomic screening of pathogenic genes in AD patients revealed that most patients (>60%) are carriers of over 10 pathogenic genes. Genes that accumulate pathogenic variants more frequently in the same patient with AD are *PRNP, PSEN1*, *ACE, A2M, BIN1*, *CLU*, *CPZ*, *LHFPL6*, *MS4A6A, MS4A4E*, and *PICALM.* Over 80% of AD patients in the Caucasian population are deficient metabolizers for the most common drugs, which are metabolized via CYP2D6, CYP2C9, CYP2C19, and CYP3A4/5 enzymes. From 60% to >90% of the patients, depending on the clinical stage of the disease, require multifactorial treatments with risk of ADRs and DDIs. The implementation of pharmacogenetics in clinical practice can help optimize the limited therapeutic resources available to treat AD and personalize the use of anti-dementia drugs in combination with other medications for the treatment of concomitant disorders.

## Figures and Tables

**Table 1:** Phenotypic features of patients with Alzheimer’s disease.

Parameter (Normal Range)	Total	Females	Males	Differences
N	2701	1491 (55%)	1210 (45%)	
Age (years) Range	67.63 ± 0.19 50–96	68.26 ± 0.27 50–96	66.86 ± 0.28 50–94	*p* < 0.001
Systolic blood pressure (mm Hg) (120–160)	141.39 ± 0.41 <120: 13.74% >160: 21.84%	141.25 ± 0.56 14.75% 22.25%	141.56 ± 0.61 12.37% 21.33%	*p* = 0.74 *p*(χ^2^) = 0.13 *p*(χ^2^) = 0.66
Diastolic blood pressure (mm Hg) (70–85)	79.38 ± 0.21 <70: 10.48% >85: 28.53%	78.83 ± 0.28 11.27% 26.84%	80.05 ± 0.33 9.54% 30.74%	*p* < 0.001 *p*(χ^2^) = 0.24 *p*(χ^2^) = 0.10
Pulse (bpm) (60–100)	68.29 ± 0.23 <60: 23.34% >100: 2.07%	69.60 ± 0.31 19.05% 2.35%	66.67 ± 0.35 28.75% 1.74%	*p* < 0.001 *p*(χ^2^) = 0.003 *p*(χ^2^) = 0.34
Weight (Kg)	72.23 ± 0.27 (36–127)	66.41 ± 0.32 (36–112)	79.41 ± 0.37 (42–127)	*p* < 0.001
Hight (cm)	160.71 ± 0.21 (120–188)	154.96 ± 0.21 (140–182)	167.79 ± 0.26 (120–188)	*p* < 0.001
BMI (Kg/m^2^) Underweight (15–18.5) Normal weight (18.5–25) Overweight (25–30) Obese Class I (moderate) (30–35) Obese Class II (severe)(35–40) Obese Class III (very severe) (>40)	29.93 ± 0.09 1.01% 25.03% 45.26% 21.25% 6.08% 1.33%	27.76 ± 0.13 1.54% 29.15% 39.50% 20.85% 7.34% 1.62%	28.15 ± 0.11 0.27% 19.86% 52.35% 21.75% 4.69% 1.08%	*p* < 0.002 *p*(χ^2^) < 0.003 *p*(χ^2^) < 0.001 *p*(χ^2^) < 0.001 *p*(χ^2^) = 0.69 *p*(χ^2^) < 0.01 *p*(χ^2^) = 0.34
Glucose (mg/dL) (70–105)	102.00 ± 0.55 <70: 0.41% >105: 26.55%	99.17 ± 0.69 0.54% 21.63%	105.49 ± 0.87 0.24% 32.70%	*p* < 0.001 *p*(χ^2^) = 0.38 *p*(χ^2^) < 0.001
Cholesterol (mg/dL) (140–220)	219.02 ± 0.90 <140: 3.60% >220: 40.54%	228.61 ± 1.22 1.81% 56.27%	207.20 ± 1.28 5.79% 21.16%	*p* < 0.001 *p*(χ^2^) < 0.001 *p*(χ^2^) < 0.001
HDL-cholesterol (mg/dL) (35–75)	55.04 ± 0.28 <35: 5.37% >75: 10.11%	59.98 ± 0.38 2.35% 15.49%	48.95 ± 0.34 9.09% 3.47%	*p* < 0.001 *p*(χ^2^) < 0.001 *p*(χ^2^) < 0.001
LDL-cholesterol (mg/dL) (80–160)	140.85 ± 0.77 <80: 5.15% >160: 29.54%	146.08 ± 1.03 3.69% 33.67%	134.41 ± 1.12 6.94% 24.46%	*p* < 0.001 *p*(χ^2^) < 0.001 *p*(χ^2^) < 0.001
Triglycerides (mg/dL) (50–150)	113.83 ± 1.31 <50: 5.59% >150: 19.44%	108.47 ± 1.50 5.97% 16.63%	120.44 ± 2.26 5.12% 22.89%	*p* < 0.001 *p*(χ^2^) = 0.41 *p*(χ^2^) < 0.001
Urea (mg/dL) (15–30)	42.90 ± 0.26 <15: 0.34% >30: 88.49%	41.79 ± 0.33 0.34% 86.45%	44.27 ± 0.40 0.34% 90.99%	*p* < 0.001 *p* = 0.75 *p*(χ^2^) = 0.37
Creatinine (mg/dL) (0.70–1.40)	0.91 ± 0.006 <0.70: 14.03% >1.40: 3.59%	0.81 ± 0.004 23.47% 1.74%	1.03 ± 0.01 2.40% 8.26%	*p* < 0.001 *p*(χ^2^) < 0.001 *p*(χ^2^) < 0.001
Uric acid (mg/dL) (3.4–7.0)	4.47 ± 0.03 <3.4: 23.77% >7.0: 6.03%	3.90 ± 0.03 36.02% 2.48%	5.17 ± 0.05 8.68% 10.41%	*p* < 0.001 *p*(χ^2^) < 0.001 *p*(χ^2^) < 0.001
Total Protein (g/dL) (6.5–8.0)	6.93 ± 0.03 <6.5: 13.77% >8.0: 1.78%	6.90 ± 0.01 12.54% 1.61%	6.97 ± 0.07 15.29% 1.98%	*p* = 0.17 *p*(χ^2^) = 0.08 *p*(χ^2^) = 0.56
Albumin (g/dL) (3.5–5.0)	4.20 ± 0.007 <3.5: 1.40% >5.0: 0.95%	4.18 ± 0.008 1.50% 0.58%	4.21 ± 0.01 1.60% 1.60%	*p* = 0.02 *p*(χ^2^) = 0.99 *p*(χ^2^) = 0.03
Calcium (mg/dL) (8.1–10.4)	9.24 ± 0.009 <8.1: 0.41% >10.4: 2.04%	9.27 ± 0.01 0.20% 2.35%	9.19 ± 0.01 0.66% 1.65%	*p* < 0.001 *p*(χ^2^) = 0.12 *p*(χ^2^) = 0.26
Phosphorus (mg/dL) (2.5–5.0)	3.41 ± 0.01 <2.5: 2.41% >5.0: 0.67%	3.52 ± 0.01 0.92% 0.81%	3.27 ± 0.01 3.47% 0.49%	*p* < 0.001 *p*(χ^2^) < 0.002 *p*(χ^2^) = 0.46
GOT/ASAT (IU/L) (10–40)	21.70 ± 0.20 <10: 0.45% >40: 3.85%	21.29 ± 0.42 0.60% 3.35%	22.20 ± 0.39 0.25% 4.46%	*p* = 0.006 *p*(χ^2^) = 0.83 *p*(χ^2^) = 0.18
GPT/ALAT (IU/L) (9–43)	23.52 ± 0.36 <9: 2.81% >43: 7.07%	21.51 ± 0.45 3.02% 4.96%	26.00 ± 0.56 2.56% 9.67%	*p* < 0.001 *p*(χ^2^) = 0.56 *p*(χ^2^) < 0.001
GGT (IU/L) (11–50)	30.55 ± 0.79 <11: 7.55% >50: 11.81%	26.37 ± 1.07 11.54% 8.52%	35.69 ± 1.17 2.65% 15.87%	*p* < 0.001 *p*(χ^2^) < 0.001 *p*(χ^2^) < 0.001
Alkaline phosphatase (IU/L) (37–111)	77.05 ± 0.62 <37: 2.07% >111: 10.37%	79.41 ± 0.85 1.74% 10.39%	74.14 ± 0.82 2.48% 10.83%	*p* < 0.001 *p*(χ^2^) = 0.24 *p*(χ^2^) = 0.98
Bilirubin (mg/dL) (0.20–1.00)	0.75 ± 0.02 <0.20: 0.30% >1.00: 15.07%	0.71 ± 0.04 0.34% 10.33%	0.80 ± 0.01 0.24% 20.91%	*p* < 0.001 *p*(χ^2^) = 0.95 *p*(χ^2^) < 0.001
CPK (IU/L) (38–174)	92.72 ± 2.00 <38: 8.22% >174: 7.29%	87.20 ± 3.12 9.25% 5.03%	99.52 ± 2.29 6.94% 10.08%	*p* < 0.001 *p*(χ^2^) < 0.05 *p*(χ^2^) < 0.001
LDH (IU/L) (200–480)	277.29 ± 1.52 <200: 13.59% >480: 1.74%	289.77 ± 2.06 6.71% 2.21%	261.88 ± 2.16 15.87% 1.16%	*p* < 0.001 *p*(χ^2^) < 0.008 *p*(χ^2^) < 0.05
Na^+^ (mEq/L) (135–148)	141.75 ± 0.04 <135: 0.70% >148: 0.89%	141.86 ± 0.05 0.36% 0.80%	141.62 ± 0.06 0.82% 0.99%	*p* < 0.002 *p*(χ^2^) = 0.65 *p*(χ^2^) = 0.76
K^+^ (mEq/L) (3.5–5.3)	4.33 ± 0.006 <3.5: 0.85% >5.3: 1.15%	4.29 ± 0.009 1.07% 0.67%	4.38 ± 0.009 0.57% 1.74%	*p* < 0.001 *p*(χ^2^) = 0.24 *p*(χ^2^) < 0.01
Cl^-^ (mEq/L) (98–107)	104.06 ± 0.07 <98: 1.22% >107: 13.92%	104.20 ± 0.13 1.07% 14.89%	103.88 ± 0.07 1.40% 12.96%	*p* < 0.04 *p*(χ^2^) = 0.55 *p*(χ^2^) = 0.29
Fe^2+^ (µg/dL) (35–160)	86.60 ± 0.70 <35: 5.04% >160: 2.61%	81.98 ± 0.89 7.01% 1.64%	92.17 ± 1.10 3.80% 4.20%	*p* < 0.001 *p*(χ^2^) < 0.01 *p*(χ^2^) < 0.001
Ferritin (ng/mL) (F: 11–307) (M: 24–336)	121.05 ± 2.91 <11: 3.43% >307: 7.65%	81.01 ± 2.48 <11: 5.55% >307: 2.09%	169.39 ± 5.32 <24: 1.8% >336: 15.3%	*p* < 0.001 *p*(χ^2^) < 0.001 *p*(χ^2^) < 0.001
Folate (ng/mL) (>3.00)	7.94 ± 0.08 <5: 27.14%	8.31 ± 0.12 23.27%	7.48 ± 0.12 31.90%	*p* < 0.001 *p*(χ^2^) < 0.001
Vitamin B_12_ (pg/mL) (170–1000)	481.98 ± 5.37 <200: 10.10%	499.87 ± 7.60 6.71%	459.99 ± 7.44 14.30%	*p* < 0.001 *p*(χ^2^) < 0.001
TSH (µIU/mL) (0.20–4.50)	1.54 ± 0.04 <0.20: 2.55% >4.50: 2.37%	1.67 ± 0.07 3.02% 2.88%	1.38 ± 0.03 1.98% 1.73%	*p* < 0.001 *p*(χ^2^) = 0.12 *p*(χ^2^) = 0.07
T4 (ng/mL) (0.54–1.40)	0.94 ± 0.01 <0.54: 0.77% >1.40: 3.15%	0.94 ± 0.02 1.07% 3.15%	0.94 ± 0.01 0.41% 3.14%	*p* = 0.55 *p*(χ^2^) = 0.08 *p*(χ^2^) = 0.92
PRL (ng/mL) (F: 1.9–25) (M: 2.5–17)	10.18 ± 0.53 <1.9: 3.48% >25: 6.53%	11.74 ± 0.89 <1.9: 2.08% >25: 6.69%	8.19 ± 0.41 <2.5: 5.17% >17: 6.32%	*p* < 0.001 *p*(χ2) < 0.03 *p*(χ2) = 0.95
Cortisol (µg/dL) (6.02–18.4)	13.46 ± 0.18 <6: 10% >18: 26.25%	13.31 ± 0.24 4.39% 17.32%	13.64 ± 0.26 3.74% 15.80%	*p* = 0.30 *p*(χ2) = 0.79 *p*(χ2) = 0.70
ACTH (pg/mL) (<46)	23.31 ± 0.62 >50: 5.25%	21.11 ± 0.78 3.69%	26.10 ± 0.99 7.18%	*p* < 0.001 *p*(χ2) < 0.05
GH (ng/mL) (F. 0.12–9.88) (M: 0.03–2.47)	0.76 ± 0.04 <0.03–0.12: 11.14% >2.47–9.80: 4.26%	0.77 ± 0.06 >0.12: 18.25% >9.80: 0.46%	0.75 ± 0.06 <0.03: 2.3% >2.47: 8.91%	*p* < 0.04 *p*(χ2) < 0.001 *p*(χ2) < 0.001
FSH (mIU/mL) (F: 21.7–153) (M: 0.7–11.1)	41.37 ± 1.36 <0.7–21.7: 4.10% >11–1–153: 11.14%	67.07 ± 1.57 <21: 6.93% >153: 0.92%	9.39 ± 0.47 <0.7: 0.57% >11: 23.85%	*p* < 0.001 *p*(χ2) < 0.001 *p*(χ2) < 0.001
LH (mIU/mL) (F: 7.7–58.5) (M: 1.7–8.6)	16.84 ± 0.53 <1.7–7.7: 6.79% >8.6–58.5: 7.30%	26.04 ± 0.63 <7.7: 7.16% >58.5: 2.77%	5.38 ± 0.20 <1.7: 6.32% >8.6: 12.93%	*p* < 0.001 *p*(χ2) = 0.77 *p*(χ2) < 0.001
Estrogen (pg/mL) (20–30)		26.23 ± 1.29 <20: 6.95% >30: 16.31%		
Testosterone (ng/dL) (193–740)			281.45 ± 11.49 <190: 31.15% >740: 1.87%	
α-Amylase (U/L) (28–100)	59.98 ± 1.26 >100: 6.70%	57.94 ± 1.64 5.70%	62.67 ± 1.95 7.77%	*p* = 0.05 *p*(χ2) = 0.43
Lipase (U/L) (13–60)	43.62 ± 0.71 >60: 10.58%	42.98 ± 0.76 9.56%	44.46 ± 1.29 11.94%	*p* = 0.75 *p*(χ2) = 0.42
AFP (ng/mL) (0–7)	3.17 ± 0.10 >7: 5.44%	3.33 ± 0.10 5.88%	2.97 ± 0.19 4.85%	*p* < 0.001 *p*(χ2) = 0.68
CEA (ng/mL) (0–3.8)	3.97 ± 1.40 >3.8: 16.76%	2.32 ± 0.09 12.99%	6.14 ± 2.26 21.75%	*p* < 0.05 *p*(χ2) < 0.01
CA 19.9 (U/mL) (0–27)	14.41 ± 2.05 >27: 8.94%	11.60 ± 0.61 7.35%	18.14 ± 4.70 11.04%	*p* = 0.27 *p*(χ2) = 0.15
CA 72.4 (U/mL) (0–6.9)	3.96 ± 0.86 >6.9: 13.01%	4.86 ± 1.46 14.71%	2.85 ± 0.66 10.91%	*p* = 0.18 *p*(χ2) = 0.78
CA 125 (U/mL) (0–35)	16.38 ± 1.67 >35: 5.97%	15.82 ± 2.42 4.19%	17.16 ± 2.14 8.50%	*p* = 0.87 *p*(χ2) = 0.16
CYFRA 21.1 (ng/mL) (0–3.3)	1.85 ± 0.08 >3.3: 10.39%	1.76 ± 0.06 9.52%	1.94 ± 0.15 11.32%	*p* = 0.83 *p*(χ2) = 0.76
SCC (ng/mL) (0–2.3)	1.30 ± 0.09 >2.3: 9.785	1.05 ± 0.05 6.59%	1.55 ± 0.18 12.96%	*p* < 0.001 *p*(χ2) = 0.11
NSE (ng/mL) (0–16.3)	10.28 ± 0.17 >16.3: 3.95%	10.77 ± 0.24 5.36%	9.78 ± 0.23 2.48%	*p* < 0.001 *p*(χ2) = 0.31
PSA (ng/mL) (<4)			2.31 ± 0.16 >4: 13.45%	
CA 15.3 (U/mL) (0–26.4)		14.05 ± 0.58 >26.4: 7.25%		
RBC (×10^6^/µL) (3.80–5.50)	4.62 ± 0.008 <3.80: 2.78% >5.50: 3.99%	4.47 ± 0.01 4.09% 2.01%	4.81 ± 0.01 1.16% 6.45%	*p* < 0.001 *p*(χ^2^) < 0.001 *p*(χ^2^) = 0.001
HCT (%) (40.0–50.0)	42.41 ± 0.23 <40.0: 29.58% >50.0: 3.07%	40.69 ± 0.26 42.85% 0.54%	44.53 ± 0.39 13.22% 6.19%	*p* < 0.001 *p*(χ^2^) = 0.001 *p*(χ^2^) = 0.001
Hb (g/dL) (13.5–17.0)	14.04 ± 0.02 <13.5: 32.06% >17.0: 2.29%	13.47 ± 0.03 46.88% 0.13%	14.74 ± 0.04 13.80% 4.95%	*p* < 0.001 *p*(χ^2^) = 0.001 *p*(χ^2^) = 0.001
MCV (fL) (80–100)	91.06 ± 0.10 <80: 2.29% >100: 3.59%	90.48 ± 0.13 2.41% 2.55%	91.77 ± 0.15 2.15% 4.88%	*p* < 0.001 *p*(χ^2^) = 0.75 *p*(χ^2^) < 0.003
MCH (pg) (27.0–33.0)	30.41 ± 0.03 <27.0: 3.62% >33.0: 5.48%	30.19 ± 0.05 4.02% 5.23%	30.69 ± 0.05 3.14% 8.26%	*p* < 0.001 *p*(χ^2^) = 0.28 *p*(χ^2^) < 0.004
MCHC (g/dL) (31.0–35.0)	33.37 ± 0.01 <31.0: 0.63% >35.0: 2.33%	33.32 ± 0.02 0.80% 2.35%	33.43 ± 0.02 0.41% 2.31%	*p* < 0.002 *p*(χ^2^) = 0.30 *p*(χ^2^) = 0.94
ADE (RDW) (%) (11.0–15.0)	12.95 ± 0.02 <11.0: 1.81% >15.0: 5.63%	13.01 ± 0.03 1.61% 6.24%	12.88 ± 0.03 2.07% 4.88%	*p* < 0.05 *p*(χ^2^) = 0.47 *p*(χ^2^) = 0.17
WBC (×10^3^/µL) (4.0–11.0)	6.35 ± 0.03 <4.0: 5.41% >11.0: 2.55%	6.18 ± 0.05 7.18% 2.28%	6.56 ± 0.05 3.47% 2.89%	*p* < 0.001 *p*(χ^2^) < 0.001 *p*(χ^2^) = 0.39
%Neu (45.0–70.0)	60.15 ± 0.19 <45.0: 6.03% >70.0: 17.92%	59.98 ± 0.25 7.04% 15.16%	60.35 ± 0.30 4.79% 16.86%	*p* = 0.22 *p*(χ^2^) < 0.02 *p*(χ^2^) = 0.33
%Lym (20.0–40.0)	29.60 ± 0.22 <20: 13.44% >40: 11.51%	30.22 ± 0.22 11.80% 12.81%	28.83 ± 0.42 15.45% 9.92%	*p* < 0.001 *p*(χ^2^) < 0.02 *p*(χ^2^) < 0.04
%Mon (3.0–10.0)	7.24 ± 0.03 <3.0: 1.33% >10.0: 9.48%	7.03 ± 0.05 1.67% 7.31%	7.50 ± 0.06 0.91% 12.15%	*p* < 0.001 *p*(χ^2^) = 0.12 *p*(χ^2^) < 0.001
%Eos (1.0–5.0)	2.81 ± 0.05 <1.0: 5.73% >5.0: 23.92%	2.61 ± 0.06 6.71% 24.21%	3.05 ± 0.09 4.55% 23.55%	*p* < 0.001 *p*(χ^2^) < 0.001 *p*(χ^2^) < 0.001
%Bas (0.0–1.0)	0.69 ± 0.07 >1.0: 14.09%	0.69 ± 0.14 9.09%	0.69 ± 0.01 19.60%	*p* < 0.001 *p*(χ^2^) < 0.001
PLT (×10^3^/µL) (150–450)	227.11 ± 1.25 <150: 7.59% >450: 0.66%	239.85 ± 1.67 4.63% 0.55%	211.29 ± 1.80 11.24% 0.85%	*p* < 0.001 *p*(χ^2^) < 0.001 *p*(χ^2^) = 0.49
MPV (fL) (6.0–10.0)	8.55 ± 0.01 <6.0: 0.18% >10.0: 9.03%	8.55 ± 0.02 0.13% 8.58%	8.55 ± 0.02 0.25% 9.58%	*p* = 0.99 *p*(χ^2^) = 0.49 *p*(χ^2^) = 0.44
MMSE Score (0–30) <25/30	23.05 ± 0.13 47.38%	22.07 ± 0.19 54.46%	24.25 ± 0.19 38.68%	*p* < 0.001 *p*(χ^2^) < 0.001
ADAS-Cog	9.37 ± 0.21	9.94 ± 0.30	8.65 ± 0.31	*p* < 0.001
ADAS-Mem	11.40 ± 0.12	11.67 ± 0.16	11.05 ± 0.17	*p* < 0.02
ADAS-Cog-T	19.06 ± 0.30	19.91 ± 0.41	18.04 ± 0.43	*p* < 0.002
ADAS-NonCog	4.75 ± 0.08	5.36 ± 0.12	3.98 ± 0.12	*p* < 0.001
ADAS-T	23.18 ± 0.36	24.66 ± 0.49	21.34 ± 0.51	*p* < 0.001
HARS <10: Normal 11–17: Mild 18–24: Mild-Moderate 25–30: Moderate-Severe	11.28 ± 0.11 39.97% 46.65% 11.36% 2.01%	12.45 ± 0.15 30.73% 51.29% 15.04% 2.94%	9.82 ± 0.15 51.37% 40.94% 6.81% 0.88%	*p* < 0.001 *p* < 0.001 *p* < 0.002 *p* < 0.001 *p* < 0.001
HDRS 0–7: Normal 8–13: Mild 14–18: Moderate 19–22: Severe >23: Very Severe	10.06 ± 0.10 34.60% 41.03% 17.18% 4.78% 2.41%	11.02 ± 0.14 28.67% 42.65% 21.84% 6.54% 3.24%	8.88 ± 0.15 42.93% 40.55% 12.19% 2.83% 1.50%	*p* < 0.001 *p* < 0.001 *p* = 0.52 *p* < 0.001 *p* < 0.001 *p* < 0.01
ECG Normal Borderline Abnormal	48.44% 10.73% 40.83%	52.10% 9.12% 38.78%	43.89% 12.74% 43.37%	*p* < 0.01 *p* < 0.01 *p* = 0.13
MRI Normal Abnormal	26.78% 73.22%	29.00% 71.00%	24.16% 75.84%	*p* = 0.52 *p* = 0.20

Data Source: CIBE DataBase (2000–2020). Statistics: Data: Mean ± SE. Normality test (Shapiro–Wilk). Equal Variance tests: Brown–Forsythe, Student’s *t* test, Welch’s *t* test. Chi Square: Fisher’s Exact test, Pearson’s Chi-Square test with Yate’s continuity correction.

**Table 2:** Frequencies of polymorphic variants in AD-related pathogenic genes.

Gene	Gene Name	OMIM	Location	dbSNP	Polymorphism	MAF	Genotype
							AA: 45.61%
*A2M*	alpha-2-macroglobulin	103,950	chr12:9067708	rs669	c.2998A>G	0.31 (G)	AG: 40.35%
							GG: 14.04%
							TT: 77.19%
*ABCA7*	ATP binding cassette subfamily A member 7	605,414	chr19:1046521	rs3764650	c.1622+115T>G	0.20 (G)	TG: 21.05%
							GG: 1.76%
							CC: 21.05%
*ACE*	angiotensin I converting enzyme	106,180	chr17:63477060	rs4332	c.496-66T>C	0.47 (T)	CT: 54.39%
							TT: 24.56%
							*2*2: <1%
							*2*3: 1.75%
*APOE*	apolipoprotein E	107,741	chr19:44908822	rs7412	c.4070C>T	0.08 (T)	*2*4: 1.75%
				rs429358	c.3932T>C	0.15 (C)	*3*3: 59.65%
							*3*4: 33.34%
							*4*4: 3.51%
							AA: 42.11%
*BIN1*	bridging integrator 1	601,248	chr2:127137039	rs744373	g.127137039A>G	0.36 (G)	AG: 50.88%
							GG: 7.01%
							CC: 59.65%
*C9ORF72*	chromosome 9 open reading frame 72	614,260	chr9:27543280	rs3849942	g.27543283T>C	0.22 (T)	TC: 36.84%
							TT: 3.51%
							GG: 36.84%
*CLU*	Clusterin	185,430	chr8:27607002	rs11136000	c.247-478A>G	0.38 (A)	AG: 49.12%
							AA: 14.04%
							CC: 17.54%
*CPZ*	carboxypeptidase Z	603,105	chr4:8650823	rs7436874	g.8649098C>T	0.36 (C)	CT: 47.37%
							TT: 35.09%
							GG: 64.92%
*CR1*	complement C3b/C4b receptor 1	120,620	chr1:207611623	rs3818361	c.4946-54A>G	0.25 (A)	AG: 29.82%
							AA: 5.26%
							TT: 94.74%
*DISC1*	disrupted in schizophrenia 1	605,210	chr1:232155150	rs16856202	c.2242-7030T>G	0.03 (G)	TG: 5.26%
							GG: <1%
							AA: 45.61%
*LHFPL6*	LHFPL tetraspan subfamily member 6	606,710	chr13:39872236	rs7995844	g.39298100G>A	0.35 (G)	GA: 38.60%
							GG: 15.79%
							CC: 49.12%
*MS4A4E*	membrane spanning 4-domains A4E	608,401	chr11:60204322	rs670139	c.279-2443C>A	0.38 (A)	CA: 42.11%
							AA: 8.77%
							CC: 36.84%
*MS4A6A*	membrane spanning 4-domains A6A	606,548	chr11:60171834	rs610932	c.*149+175A>C	0.45 (A)	CA: 38.60%
							AA: 24.56%
							GG: 50.88%
*NOS3*	nitric oxyde synthse 3	163,729	chr7:150991055	rs1799983	c.894G>T	0.18 (T)	GT: 35.09%
							TT: 14.03%
							CC: 45.61%
*PICALM*	phosphatidylinositol binding clathrin assembly protein	603,025	chr11:86157598	rs3851179	g.85868640T>C	0.31 (T)	CT: 42.11%
							TT: 12.28%
							GG: 19.30%
*PRNP*	prion protein	176,640	chr20:4686093	rs1799990	c.385A>G	0.73 (A)	AG: 40.35%
							AA: 40.35%
							TT: 22.80%
*PSEN1*	presenilin 1	104,311	chr14:73136434	rs165932	c.856+16G>T	0.43 (G)	GT: 35.09%
							GG: 42.11%
							GG: 73.68%
*TNF*	tumor necrosis factor	191,160	chr6:31575566	rs1800629	c.-308G>A	0.09 (A)	GA: 22.81%
							AA: 3.51%

**Table 3:** Frequencies of polymorphic variants in cerebrovascular disease-related genes in Alzheimer’s disease.

Gene Symbol	Gene Name	OMIM	Location	dbSNP ID	Polymorphism	MAF	Genotype
							CC: 18.70%
*ACE*	angiotensin I converting enzyme	106,180	chr17:63486920	rs4332	c.496-66T>C	0.47 (T)	CT: 39.81%
							TT: 41.49%
							CC: 11.46%
*AGT*	Angiotensinogen	106,150	chr1:230710231	rs4762	c.620C>T	0.10 (T)	CT: 21.96%
							TT: 66.58%
							TT: 21.97%
*AGT*	Angiotensinogen	106,150	chr1:230710048	rs699	c.803T>C	0.30 (T)	TC: 56.48%
							CC: 21.61%
							CC: 29.03%
*APOB*	apolipoprotein B	107,730	chr2:21009323	rs693	c.2488C>T	0.25 (T)	CT: 47.64%
							TT: 23.33%
							CC: 78.94%
*APOC3*	apolipoprotein C-III	107,720	chr11:116832924	rs5128	c.3175C>G	0.23 (C)	CG: 17.60%
							GG: 3.46%
							*2*2: 0.32%
							*2*3: 7.62%
*APOE*	apolipoprotein E	107,741	chr19:44908822	rs7412	c.4070C>T	0.08 (T)	*2*4: 1.28%
			chr19:44908684	rs429358	c.3932T>C	0.15 (C)	*3*3: 63.73%
							*3*4:23.88%
							*4*4: 3.17%
							GG: 37.39%
*CETP*	cholesteryl ester transfer protein	118,470	chr16:56962376	rs708272	c.+279G>A	0.38 (A)	GA: 49.42%
							AA: 13.19%
							GG: 96.41%
*F2*	coagulation factor II, thrombin	176,930	chr11:46739505	rs1799963	c.20210G>A	0.01 (A)	GA: 3.47%
							AA: 0.12%
							GG: 98.02%
*F5*	coagulation factor V	227,400	chr1:169549811	rs6025	c.1691G>A	0.01 (A)	GA: 1.61%
							AA: 0.37%
							TT: 4.59%
*IL1B*	interleukin 1 beta	147,720	chr2:112832813	rs1143634	c.3954T>C	0.13 (T)	TC: 31.39%
							CC: 64.02%
							GG: 39.95%
*IL6*	interleukin 6	147,620	chr7:22727026	rs1800795	c.-174G>C	0.14 (C)	GC: 43.55%
							CC: 16.50%
							GG: 81.12%
*IL6*	interleukin 6	147,620	chr7:22726627	rs1800796	c.-573G>C	0.31 (C)	GC: 15.90%
							CC: 2.98%
							AA: 34.41%
*IL6R*	interleukin 6 receptor	147,880	chr1:154454494	rs2228145	c.1510A>C	0.36 (C)	AC: 49.69%
							CC: 15.90%
							CC: 76.02%
*LPL*	lipoprotein lipase	609,708	chr8:19962213	rs328	c.1421C>G	0.09 (G)	CG: 20.00%
							GG: 3.98%
							CC: 38.90%
*MTHFR*	methylenetetrahydrofolate reductase	607,093	chr1:11796321	rs1801133	c.665C>T	0.25 (T)	CT: 45.84%
							TT: 15.26%
							AA: 50.36%
*MTHFR*	methylenetetrahydrofolate reductase	607,093	chr1:11794419	rs1801131	c.1286A>C	0.25 (C)	AC: 39.90%
							CC: 9.74%
							GG: 39.54%
*NOS3*	nitric oxyde synthse 3	163,729	chr7:150991055	rs1799983	c.894G>T	0.18 (T)	GT: 47.67%
							TT: 12.79%
							GG: 73.79%
*TNF*	tumor necrosis factor	191,160	chr6:31575566	rs1800629	c.-308G>A	0.09 (A)	GA: 22.86%
							AA: 3.35%

**Table 4:** Pharmacological properties and pharmacogenetics of conventional anti-dementia drugs.

Drug	Properties	Pharmacogenetics
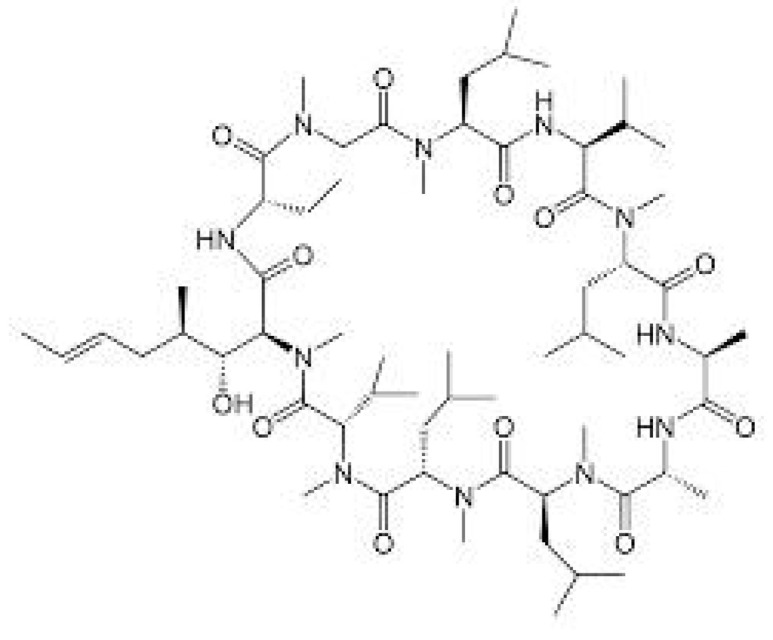	**Name:Aducanumab,** BIIB-037, Aduhelm, 1384260-65-4 IUPAC Name: Immunoglobulin G1, anti-(human.beta.-amyloid) (human monoclonal biib037 heavy chain), disulfide with human monoclonal biib037.kappa.-chain, and dimer **Molecular Formula:** C_6472_H_10028_N_1740_O_2014_S_46_ **Molecular Weight:** 145910.3123 g/mol **Category:** Monoclonal antibody (mAb), anti-amyloid beta A4 protein **Mechanism:** Monoclonal IgG1 antibody that binds to amyloid-β, reducing amyloid plaques in the brain **Effect:** Anti-amyloid beta A4 protein slowing the rate of progression of Alzheimer’s disease and levels of p-tau in the cerebrospinal fluid	**Pathogenic genes:** *APP, APOE*, *PSEN1, PSEN2* **Mechanistic genes:** **Drug metabolism-related genes:** **-Substrate:** **-Inhibitor:** **Transporter genes:** **Pleiotropic genes:** *APOE, IL6, IL1B, TNF*
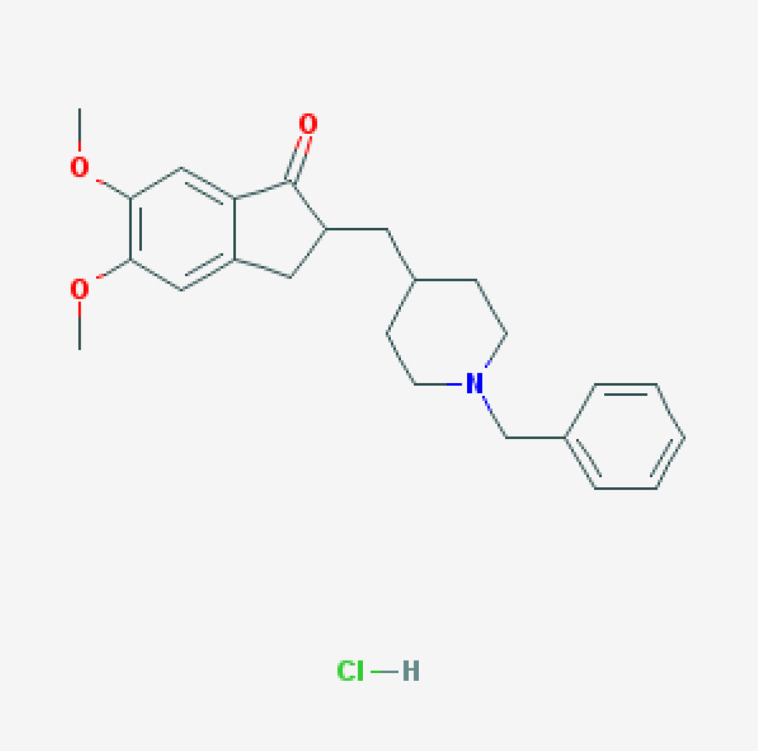	**Name**: **Donepezil hydrochloride,** Aricept, 120011-70-3, Donepezil HCl, BNAG, E-2020, and E2020 **IUPAC Name:** 2-[(1-benzylpiperidin-4-yl)methyl]-5,6-dimethoxy-2,3-dihydroinden-1-one;hydrochloride **Molecular Formula:** C_24_H_30_ClNO_3_ **Molecular Weight:** 415.9529 g/mol **Category:** Cholinesterase inhibitor **Mechanism:** Centrally active, reversible acetylcholinesterase inhibitor; increases the acetylcholine available for synaptic transmission in the CNS **Effect:** Nootropic agent, cholinesterase inhibitor, and parasympathomimetic effect	**Pathogenic genes:** *APP, APOE*, *CHAT* **Mechanistic genes:** *ACHE*, *BCHE, CHAT*, *CHRNA7* **Drug metabolism-related genes:** **-Substrate:** *CYP2D6* (major), *CYP3A4* (major), *UGTs*, *ACHE* **-Inhibitor:** *ABCB1, ACHE*, *BCHE, hERG* **Transporter genes:** *ABCB1, ABCA1, ABCG2, SCN1A* **Pleiotropic genes:** *APOE, PLP, MAG, MBP, CNPase, MOG*
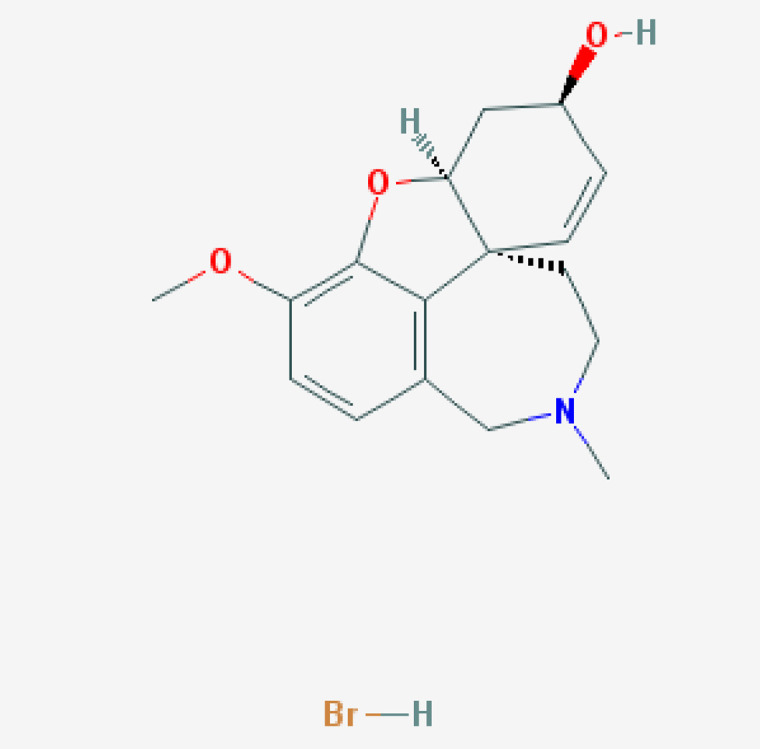	**Name**: **Galantamine hydrobromide:** Galanthamine hydrobromide, 1953-04-4, Nivalin, Razadyne, UNII-MJ4PTD2VVW, and Nivaline **IUPAC Name:** (1S,12S,14R)-9-methoxy-4-methyl-11-oxa-4-azatetracyclo [8.6.1.0^{1,12}.0^{6,17}]heptadeca-6,8,10(17),15-tetraen-14-ol **Molecular Formula:** C_17_H_22_BrNO_3_ **Molecular Weight:** 368.26548 g/mol **Category:** Cholinesterase inhibitor **Mechanism:** Reversible and competitive acetylcholinesterase inhibition leading to an increased concentration of acetylcholine at cholinergic synapses; modulates nicotinic acetylcholine receptor; may increase glutamate and serotonin levels **Effect:** Nootropic agent, cholinesterase inhibitor, and parasympathomimetic effect	**Pathogenic genes:** *APOE, APP* **Mechanistic genes:** *ACHE, BCHE, CHRNA4, CHRNA7, CHRNB2, SLC18A3* **Drug metabolism-related genes:** **-Substrate:** *ABCB1, CYP2D6* (major), *CYP3A4* (major), *UGT1A1* **-Inhibitor:** *ACHE, BCHE* **Transporter genes:** *ABCB1, SLC18A3*
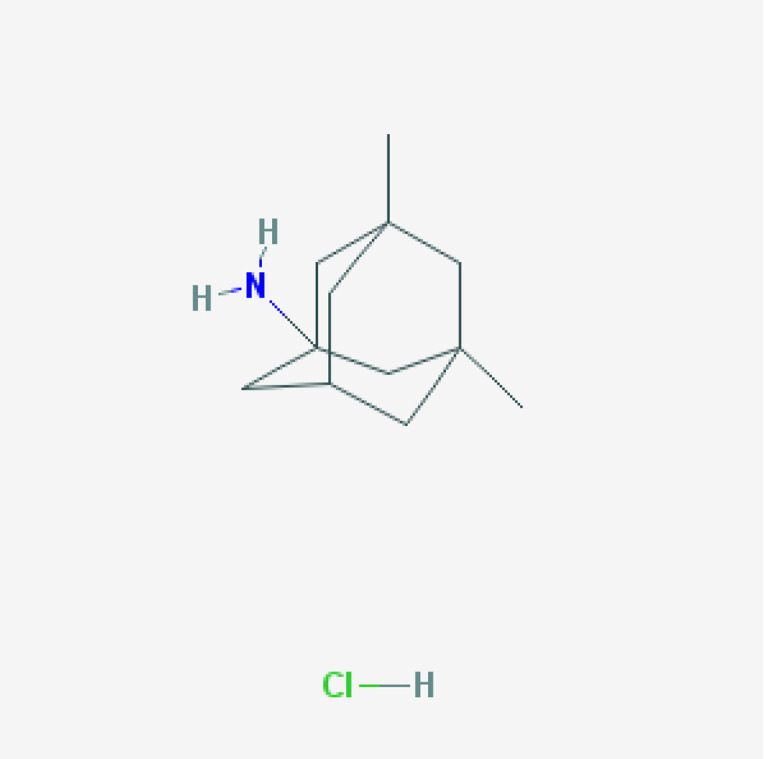	**Name**: **Memantine Hydrochloride,** 41100-52-1, Namenda, Memantine HCL, Axura, 3,5-Dimethyl-1-adamantanamine hydrochloride, and 3,5-dimethyladamantan-1-amine hydrochloride **IUPAC Name:** 3,5-dimethyladamantan-1-amine;hydrochloride **Molecular Formula:** C_12_H_22_ClN **Molecular Weight:** 215.76278 g/mol **Category:** N-Methyl-D-Aspartate receptor antagonist **Mechanism:** Binds preferentially to NMDA receptor-operated cation channels; may act by blocking actions of glutamate, mediated in part by NMDA receptors **Effect:** Dopamine agent, antiparkinson agent, excitatory amino acid antagonist, and antidyskinetic	**Pathogenic genes:** *APOE, MAPT, PSEN1* **Mechanistic genes:** *CHRFAM7A, DLGAP1, FOS, GRIN2A, GRIN2B, GRIN3A, HOMER1, HTR3A* **Drug metabolism-related genes:** **-Inhibitor:** *CYP1A2* (weak), *CYP2A6* (weak), *CYP2B6* (strong), *CYP2C9* (weak), *CYP2C19* (weak), *CYP2D6* (strong), *CYP2E1* (weak), *CYP3A4* (weak), *NR1I2* **Transporter genes:** *NR1I2* **Pleiotropic genes:** *APOE, MAPT, MT-TK, PSEN1*
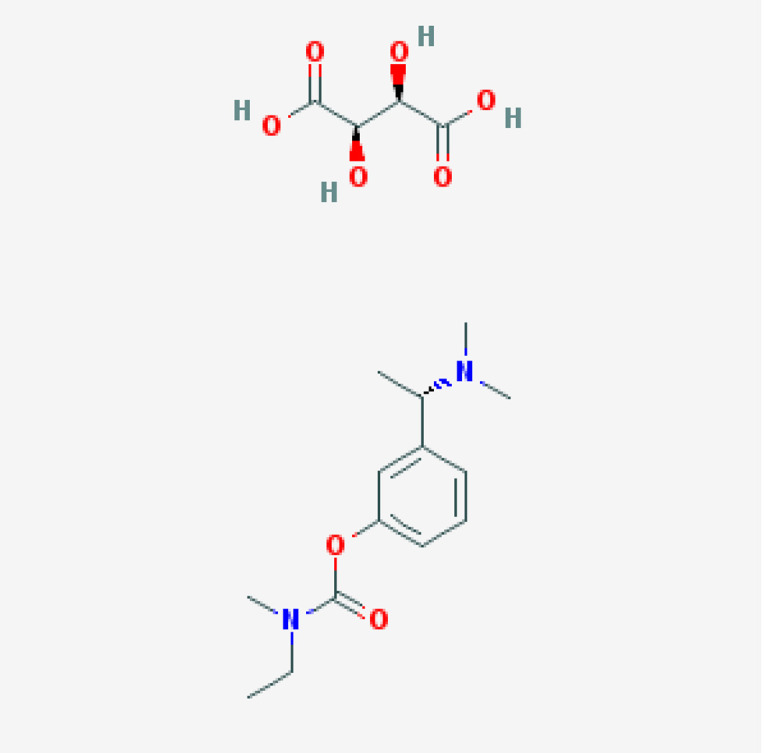	**Name**: **Rivastigmine tartrate,** 129101-54-8, SDZ-ENA 713, Rivastigmine hydrogentartrate, Rivastigmine Hydrogen Tartrate, ENA 713, and ENA-713 **IUPAC Name:** (2R,3R)-2,3-dihydroxybutanedioic acid;[3-[(1S)-1-(dimethylamino)ethyl]phenyl] N-ethyl-N-methylcarbamate **Molecular Formula:** C_18_H_28_N_2_O_8_ **Molecular Weight:** 400.42352 g/mol **Category:** Cholinesterase inhibitor **Mechanism:** Increases acetylcholine in CNS through reversible inhibition of its hydrolysis by acetylcholinesterase **Effect:** Neuroprotective agent, cholinesterase inhibitor, and cholinergic agent	**Pathogenic genes:** *APOE, APP, CHAT* **Mechanistic genes:** *ACHE, BCHE, CHAT, CHRNA4, CHRNB2, SLC18A3* **Drug metabolism-related genes:** **-Substrate:** *UGT1A9, UGT2B7* **-Inhibitor:** *ACHE, BCHE* **Transporter genes:** *SLC18A3* **Pleiotropic genes:** *APOE, MAPT*
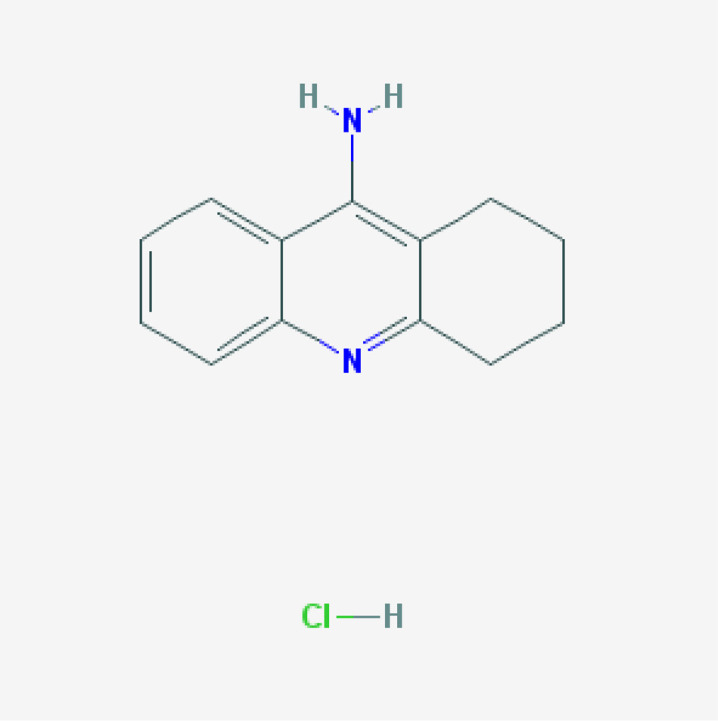	**Name**: **Tacrine Hydrochloride,** Tacrine HCl, 1684-40-8, Hydroaminacrine, tacrine.HCl, 9-AMINO-1,2,3,4-TETRAHYDROACRIDINE HYDROCHLORIDE, and Tenakrin **IUPAC Name:** 1,2,3,4-tetrahydroacridin-9-amine;hydrochloride **Molecular Formula:** C_13_H_15_ClN_2_ **Molecular Weight:** 234.7246 g/mol **Category:** Cholinesterase inhibitor **Mechanism:** Elevates acetylcholine in cerebral cortex by slowing degradation of acetylcholine **Effect:** Nootropic agent, cholinesterase inhibitor, and parasympathomimetic effect	**Pathogenic genes:** *APOE* **Mechanistic genes:** *ACHE, BCHE, CHRNA4, CHRNB2* **Drug metabolism-related genes:** **-Substrate:** *CYP1A2 (*major*), CYP2D6 (*minor*), CYP3A4 (*major*), CES1, GSTM1, GSTT1* **-Inhibitor:** *ACHE, BCHE, CYP1A2* (weak) **Transporter genes:** *ABCB4, SCN1A* **Pleiotropic genes:** *APOE, LEPR, MTHFR*
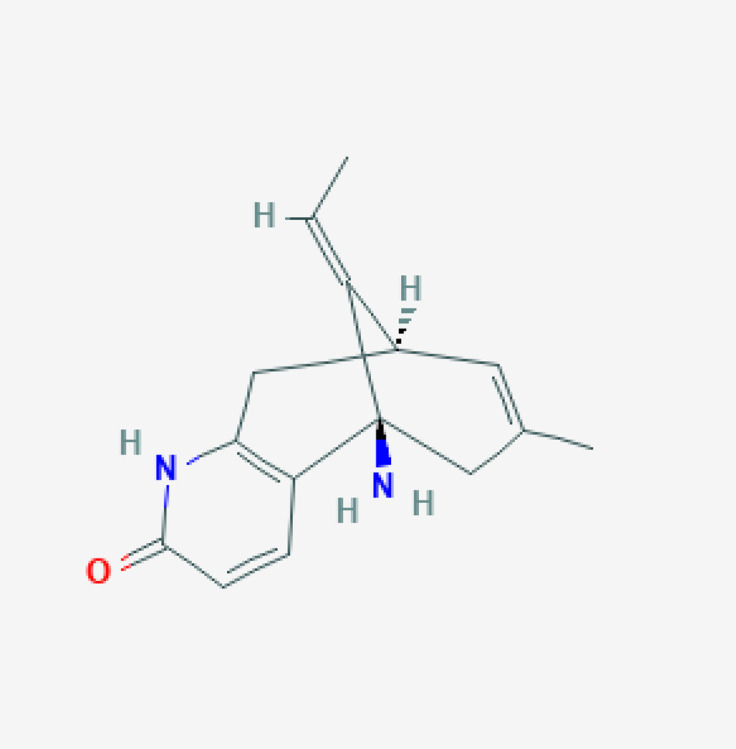	**Name**: **(-)-Huperazine A,** Huperzine A; Huperzine-A; 102518-79-6; (-)-Huperzine A; (+/−)-Huperzine A **IUPAC name:** (1R,9R,13E)-1-amino-13-ethylidene-11-methyl-6-azatricyclo[7.3.1.02,7]trideca-2(7),3,10-trien-5-one **Molecular Formula:** C_15_H_18_N_2_O **Molecular Weight:** 242.32g/mol **Category:** Neuroprotectanct, Cholinesterase Inhibitor **Mechanism:** Increases acetylcholine in the brain by inhibiting acetylcholinesterase and slowing acetylcholine hydrolysis **Effect:** Neuroprotective, acetylcholinesterase inhibitor, cognitive enhancer, and antiepileptic	**Pathogenic genes:** *APP, APOE* **Mechanistic genes:** *ACHE* **Drug metabolism-related genes:** **-Substrate:** *ABCB1, CYP1A2, CYP3A1, CYP3A2, CYP2C11, CYP2E1, CES1, CES2* **-Inhibitor:** *ACHE* **-Inducer:** *CYP1A2* **Transporter genes:** *ABCB1, ABCG2* **Pleiotropic genes:** *APOE, BDNF*

**ABCA1**: ATP-Binding Cassette, Subfamily A, Member 1; **ABCB1:** ATP-Binding Cassette, Subfamily B, Member 1; **ABCB4:** ATP-Binding Cassette, Subfamily B, Member 4; **ABCG2**: ATP-Binding Cassette, Subfamily G, Member 2; **ACHE**: Acetylcholinesterase; **APOE**: Apolipoprotein E; **APP**: Amyloid Precursor Protein; **BCHE**: Butyrylcholinesterase; BDNF: Brain-derived neurotrophic factor; **CES1**: Carboxylesterase 1; **CES2**: Carboxylesterase 2; **CHAT**: Cholineacetyltransferase; **CHRNA4**: Cholinergic Receptor, Neuronal Nicotinic, Alpha Polypeptide 4; **CHRNA7**: Cholinergic Receptor, Neuronal Nicotinic, Alpha Polypeptide 7; **CHRNB2**: Cholinergic receptor nicotinic beta 2 subunit; **CNPase**: Cyclic Nucleotide Phosphodiesterase; **CYP1A2**: Cytochrome P450, family 1, subfamily A, polypeptide 2; **CYP2A6**: Cytochrome P450, family 2, subfamily A, polypeptide 6; **CYP2B6**: Cytochrome P450, family 2, subfamily B, polypeptide 6; **CYP2C1**: Cytochrome P450, family 2, subfamily C, polypeptide 1; **CYP2C9**: Cytochrome P450, family 2, subfamily C, polypeptide 9; **CYP2C11**: Cytochrome P450, family 2, subfamily C, polypeptide 11; **CYP2C19**: Cytochrome P450, family 2, subfamily C, polypeptide 19; **CYP2D6**: Cytochrome P450, family 2, subfamily D, polypeptide 6; **CYP2E1**: Cytochrome P450, family 2, subfamily E, polypeptide 1; **CYP3A1**: Cytochrome P450, family 3, subfamily A, polypeptide 1; **CYP3A2**: Cytochrome P450, family 3, subfamily A, polypeptide 2; **CYP3A4**: Cytochrome P450, family 3, subfamily A, polypeptide 4; **DLGAP1**: discs, large (Drosophila) homolog-associated protein 1; **FOS:** FBJ murine osteosarcoma viral oncogene homolog; **GRIN2A**: Glutamate receptor, ionotropic, N-methyl-D-aspartate, subunit 2A; **GRIN2B**: Glutamate receptor, ionotropic, N-methyl-D-aspartate, subunit 2B; **GRIN3A**: Glutamate receptor, ionotropic, N-methyl-D-aspartate, subunit 3A; **GSTM1**: Glutathione S-transferase mu 1; **HOMER1:** homer homolog 1 (Drosophila); **HTR3:** 5-hydroxytryptamine receptor 3; **IL1B:** Interleukin 1 beta; **IL6:** Interleukin 6; **LEPT:** Leptin receptor; **MAPT**: Microtubule-Associated Protein Tau; **MBP**: Myelin basic protein; **MOG**: Myelin-oligodendrocyte glycoprotein; **MTHFR**: 5,10-Methylenetetrahydrofolate reductase; **NR1I2**: nuclear receptor subfamily 1, group I, member 2; **PLP**: Proteolipid protein; **PSEN1**: Presenilin 1; **PSEN2**: Presenilin 2; **SCN1A**: Sodium voltage-gated channel, alpha subunit 1; **SLC18A3**: Solute Carrier Family 18 (Vesicular Acetylcholine), Member 3; **UGT1A1**: UDP glucuronosyltransferase 1 family, polypeptide A1; **UGT1A9**: UDP glucuronosyltransferase 1 family, polypeptide A9; **UGT2B7**: UDP glucuronosyltransferase 2 family, polypeptide B7; and **TNF**: Tumor necrosis factor.

## Data Availability

Data supporting reported results can be found in the CIBE Database at EuroEspes International Center of Neuroscience and Genomic Medicine. www.euroespes.com (accessed on 3 February 2022).

## Abbreviations

ABCB1: ATP binding cassette subfamily B member 1; ABCC2: ATP binding cassette subfamily C member 2; ABCG2: ATP binding cassette subfamily G member 2 (Junior blood group); ADRA2A: adrenoceptor alpha 2A; ADRB2: adrenoceptor beta 2; ADRB3: adrenoceptor beta 3; AGT: angiotensinogen; APOE: apolipoprotein E; BCHE: butyrylcholinesterase; CES1: carboxylesterase 1; CHAT: choline O-acetyltransferase; CHRNA7: cholinergic receptor nicotinic alpha 7 subunit; COMT: catechol O-methyltransferase; CYP1A1: cytochrome P450 family 1 subfamily A member 1; CYP1A2: cytochrome P450 family 1 subfamily A member 1; CYP1B1: cytochrome P450 family 1 subfamily B member 1; CYP2A6: cytochrome P450 family 2 subfamily A member 6; CYP2B6: cytochrome P450 family 2 subfamily B member 6; CYP2C19: cytochrome P450 family 2 subfamily C member 19; CYP2C9: cytochrome P450 family 2 subfamily C member 9; CYP2D6: cytochrome P450 family 2 subfamily D member 6; CYP2E1: cytochrome P450 family 2 subfamily E member 1; CYP3A4: cytochrome P450 family 3 subfamily A member 4; CYP3A5: cytochrome P450 family 3 subfamily A member 5; CYP4F2: cytochrome P450 family 4 subfamily F member 2; DPYD: dihydropyrimidine dehydrogenase; DRD2: dopamine receptor D2; FABP2: fatty acid binding protein 2; G6PD: glucose-6-phosphate dehydrogenase; GABRA1: gamma-aminobutyric acid type A receptor alpha1 subunit; GSTM1: glutathione S-transferase mu 1; GSTP1: glutathione S-transferase pi 1; GSTT1: glutathione S-transferase theta 1; HMGCR: 3-hydroxy-3-methylglutaryl-CoA reductase; HTR2A: 5-hydroxytryptamine receptor 2A; HTR2C: 5-hydroxytryptamine receptor 2C; IFNL3: interferon lambda 3; MAOB: monoamine oxidase B; MTHFR: methylenetetrahydrofolate reductase; NAT2: N-acetyltransferase 2; NBEA: neurobeachin: OPRM1: mu-type opioid receptor; PPARG: peroxisome proliferator activated receptor gamma; PRKCE: protein kinase C epsilon; PTGS2: prostaglandin-endoperoxide synthase 2; RYR1: ryanodine receptor 1; SLC22A1: solute carrier family 22 member 1; SLC2A2: solute carrier family 2 member 2; SLC2A9: solute carrier family 2 member 9; SLC30A8: solute carrier family 30 member 8; SLC39A8: solute carrier family 39 member 8; SLC6A2: solute carrier family 6 member 2; SLC6A3: solute carrier family 6 member 3; SLC6A4: solute carrier family 6 member 4; SLCO1B1: solute carrier organic anion transporter family member 1B1; SOD2: superoxide dismutase 2; SOD3: superoxide dismutase 3; TPMT: thiopurine S-methyltransferase; UGT1A1: UDP glucuronosyltransferase family 1 member A1; VKORC1: vitamin K epoxide reductase complex subunit 1.
